# Synthesis challenges in complex evidence: A critical analysis of systematic reviews of face mask efficacy

**DOI:** 10.1017/rsm.2026.10072

**Published:** 2026-02-06

**Authors:** Trisha Greenhalgh, Sahanika Ratnayake, Rebecca Helm, Luana Poliseli, Jon Williamson

**Affiliations:** 1 Nuffield Department of Primary Care Health Sciences, University of Oxford, UK; 2 Philosophy, University of Manchester – The Victoria University of Manchester Campus, UK; 3 Law, University of Exeter, UK

**Keywords:** case control studies, causal inference, cohort studies, general methods, medicine, qualitative research

## Abstract

The evaluation of the role of face masks in preventing respiratory infections is a paradigm case in synthesising complex evidence (i.e. extensive, diverse, technically specialised, and with multilevel chains of causality). Primary studies have assessed different mask types, diseases, populations, and settings using different research designs. Numerous review teams have attempted to synthesise this literature, in which observational (case–control, cohort, cross-sectional) and ecological studies predominate. Their findings and conclusions vary widely.

This article critically examines how 66 systematic reviews dealt with mask efficacy studies. Risk-of-bias tools produced unreliable assessments when—as was often the case—review teams lacked methodological expertise or topic-specific understanding. This was especially true when datasets were large and heterogeneous, with multiple biases playing out in different ways and requiring nuanced adjustments. In such circumstances, tools were sometimes used crudely and reductively rather than to support close reading of primary studies and guide expert judgments. Various moves by reviewers—excluding observational evidence altogether, assessing risk but not direction of biases, omitting distinguishing details of primary studies, and producing meta-analyses that combined studies of different designs or included studies at critical risk of bias—served to obscure important aspects of heterogeneity, resulting in bland and unhelpful summary statements.

We draw on philosophy to question the formulaic use of generic risk-of-bias tools, especially when the primary evidence demands expert understanding and tailoring of study quality questions to the topic. We call for more rigorous training and oversight of reviewers of complex evidence and for new review methods designed specifically for such evidence.

## Highlights

### What is already known?

- 66 systematic reviews of face mask efficacy reached different conclusions
- Most mask efficacy studies are observational

### What is new?

- Risk-of-bias tools were sometimes misapplied in non-expert hands
- The review process sometimes obscured important aspects of heterogeneity, leading to bland and unhelpful summary statements
- Many review teams lacked topic expertise, methods expertise or both

### Potential impact for RSM readers

- The claim of ‘PRISMA-compliance’ may obscure questionable practices and judgements
- Training and oversight of reviewers is crucial when primary evidence is complex (i.e. extensive, heterogeneous, technically specialised, conflicting, contested)

## 1 Background

### 1.1 Introduction

The evidence base on some scientific issues is complex—large in volume, heterogeneous in focus and study design, and addressing complex chains of causality at different levels from molecular interactions to human behaviour to societal contexts. Mask efficacy in preventing respiratory infections is one such example. In an ongoing study of evidence synthesis methods related to this topic,^1^ we have identified approximately 300 primary studies on mask efficacy, of which only 26 are randomised controlled trials (RCTs). Some of these primary studies were carefully planned and rigorously executed; others occurred opportunistically during crisis periods. These studies are summarised in (so far) 66 systematic reviews, which addressed a variety of review questions using different approaches and came to seemingly opposing conclusions.

The predominance of observational (non-RCT) studies on the topic of mask efficacy raises the question of how (if at all) systematic reviews should include such designs. Several recent systematic reviews—notably, an updated Cochrane review^2^—have chosen to reject all observational studies of mask efficacy outright. Others believe that there are over-riding practical and ethical difficulties associated with conducting RCTs of mask efficacy and that even when internally valid (i.e. methodologically rigorous), a mask RCT may lack external validity (findings from Bangladesh villages during low-prevalence periods, for example,^3^ do not tell us what will happen in other settings).^4^ ^,^ ^5^ A previous commentary from Cochrane editors on systematic reviews of public health interventions concluded that the vast amount of available observational evidence should, with appropriate quality assessments and caveats, be included in evidence syntheses to enrich the debate.^6^

Observational studies of mask efficacy present challenges for reviewers because they are inconsistently titled, heterogeneous in study design and methodology, variable in quality, dispersed across a range of non-mainstream journals (since most leading journals favour RCT designs), and poorly indexed.^7^ A further challenge is the sheer volume of such evidence, which means that there is a high risk that studies will be read superficially or partially (e.g. with a view to populating predefined data fields) rather than fully and in depth.

Mask efficacy thus presents a paradigm case in how reviewers might handle complex primary evidence, especially heterogeneous datasets of observational studies. In this article, we critically examine how such studies have been evaluated by systematic reviewers and meta-analysts.

The remainder of this article is structured as follows. In the next section, we explain what we mean by ‘observational evidence’ in the context of mask efficacy and briefly summarise the strengths and limitations of different study designs. We then review orthodox methods of evidence synthesis for observational studies before introducing our aims and research questions. In the Methods section, we describe how we identified and critically examined a dataset of primary studies and the systematic reviews that had attempted to synthesise them. In the Results section, we describe how variation in search strategies, eligibility criteria, and synthesis methods led review teams to work with widely differing datasets of primary studies and generate different estimates of efficacy (and inefficacy). In the Discussion section, we question how far generic evidence synthesis tools can contribute to reviews when the primary evidence demands expert understanding and tailoring of study quality questions to the topic.

### 1.2 Observational studies of mask efficacy: an overview

Four broad kinds of observational study have addressed mask efficacy.

A *case–control study* is retrospective. It compares people with a disease (e.g. those testing positive for COVID-19) with those without, with a view to finding associations with past exposures (e.g. being unmasked when mixing with potentially infectious people). In a *matched* case–control design, cases are individually paired with controls who are similar in key characteristics that could influence both mask-wearing and disease risk. In an *unmatched* case–control design, individual matching is not used, and comparability at the group level is relied upon instead. Differences between groups are usually measured using odds ratio (OR), a measure of the relative odds of having been exposed, given that one has the disease, compared with not having the disease. Case–control studies are quick and relatively cheap to perform, but their retrospective nature makes them highly susceptible to recall and selection biases. Hence, any associations demonstrated in such studies may or may not be causal, and the measured effect size (or lack of it) may be an over- or underestimate.

In *prospective cohort* studies, two groups differing in exposure (e.g. masking or not during an infectious disease outbreak) are followed forward in time to see whether they differ in the incidence (new cases) of a disease. Differences between groups are usually measured using risk ratio (RR), a measure of how likely someone in one group is to develop the disease compared with someone in the other group. Risk difference (RD), which measures the absolute difference in risk between the exposed and unexposed groups, may also be used. Prospective cohort designs provide a stronger basis for inferring causality than case–control studies, but they are more expensive and time-consuming to conduct and face significant challenges in controlling for complex behavioural confounders (e.g. whether people who mask also differ systematically in how often they wash their hands, mix indoors with other people, or smoke). Cohort studies can also be undertaken retrospectively, with a greater risk of bias.

In *cross-sectional* studies, exposure (e.g. a response to a survey question about masking) and outcome (e.g. a swab for current infection or a test for antibodies to a disease) are measured at a single point in time. Such studies can compare disease prevalence in those who have or have not been masking during a recent outbreak. Differences between groups are measured in terms of prevalence. While cross-sectional studies can establish associations, they provide a weaker basis for inferring causality than either case–control or cohort studies, since the absence of a longitudinal component means a putative causal factor may or may not have predated the onset of disease.

*Ecological studies* (sometimes known as ‘natural experiments’) can trace what happens when a policy (e.g. a mask mandate) is introduced in a particular defined locality (e.g. a country, state, or city). These studies may use mathematical modelling to compute the complex interaction between real-world variables over time. Study designs range from simple before-and-after studies in a single setting, analysed using descriptive statistics (e.g. interrupted time series) through designs comparing one setting with another (mandate in place versus mandate not in place using difference-in-difference, regression, or synthetic controls) to mechanistic modelling (e.g. using compartmental SEIR—susceptible, exposed, infected, recovered—models). Ecological studies are sometimes included in the category ‘observational studies’, but they are usually excluded from systematic reviews of observational evidence on the assumption that multiple confounders are in play, and hence, grounds for causal inference are weak.

### 1.3 Synthesising observational evidence: tools for quality assessment

Observational evidence lacks the neatness of a controlled experiment, is vulnerable to numerous biases, and tends to be heterogeneous in multiple ways. The challenges this poses for evidence synthesis include the perils of spurious precision if meta-analysis is used to combine irregular primary evidence; arguments for and against the *automatic* downgrading of observational studies relative to RCTs (meaning that a high-quality observational study will be ranked as equivalent to, or even as lower than, a poor-quality RCT); and how to evaluate researchers’ efforts to mitigate the biases inherent in non-randomised designs.^6^ ^,^ ^8^ ^–^ ^10^

Tools to guide readers in assessing the quality of published research studies emerged in the late 1990s. The first such tools were described as ‘critical appraisal checklists’, of which early examples for observational studies include those from the Critical Appraisal Skills Programme (CASP) in the UK^11^ and the Joanna Briggs Institute (JBI) in Australia (shown in Table 1).^12^ Another early tool was the Newcastle-Ottawa [Quality Assessment] Scale (NOS),^13^ also shown in Table 1 and described by its authors as a ‘quality assessment tool’.

**Table 1:** Tools used for assessing quality in observational studies Table 1 Long description.

Described by the authors as	Questions or domains	Scoring
Joanna Briggs institute checklist for aetiology and risk (first published 2003)^12^		
Critical appraisal checklist	Were groups comparable other than presence / absence of disease in cases / controls? Were cases and controls matched appropriately? Were the same criteria used for identifying cases and controls? Was exposure measured in a standard, valid and reliable way? Was exposure measured in the same way for cases and controls? Were confounding factors identified? Were strategies to deal with confounding factors stated? Were outcomes assessed in a standard, valid and reliable way for cases and controls? Was the exposure period of interest long enough to be meaningful? Was appropriate statistical analysis used?	Each question is scored ‘yes’ (criterion met), ‘no’ (criterion not met), ‘unclear’ or ‘not applicable’. Overall score calculated by summing all domains.
Newcastle-Ottawa scale for case–control studies (first published 1999)^13^		
Quality assessment tool	Selection Adequacy of case definition Representativeness of cases Selection of controls Definition of controls Comparability Key confounders controlled for? Exposure How exposure was ascertained Same for cases and controls? Losses to follow-up similar in both groups?	Maximum of 9 stars can be awarded across all domains (domain 5 can have up to 2 stars). Overall score is sum of all domains. Scores can be collapsed as 0–3 ‘low [quality]’, 4–6 ‘medium’, 7–9 ‘high’.
Robins-I (risk of bias in non-randomised studies of interventions, first published 2016)^16^		
Risk of bias tool for causal inference	Confounding (‘case-mix bias’).^a^ Selection of participants (‘selection bias’).^b^ Classification of interventions (‘misclassification bias’, ‘measurement bias’, ‘recall bias’).^c^ Deviations from intended interventions (‘performance bias’).^d^ Missing data (‘attrition bias’).^e^ Measurement of outcomes (‘detection bias’).^f^ Selection of reported results (‘reporting bias’).^g^	Risk of bias in each domain rated ‘low’, ‘moderate’, ‘serious,’ or ‘critical’. Lowest-scoring domain determines the overall risk of bias.
Grade (certainty of evidence) criteria for non-randomised studies (first published 2011)		
Guideline development framework	Risk of bias (e.g. using ROBINS-I). Inconsistency (e.g. unexplained heterogeneity). Indirectness (i.e. external validity). Imprecision (e.g. small effect size with large confidence interval). Other considerations (e.g. higher certainty if large effect size, dose-response relationship, and if residual confounding would bias result toward the null).	Grade each domain ‘high’, ‘moderate’, ‘low’ or ‘very low’ certainty. ‘High’ = high confidence that true effect is close to the estimate.

Notes on Table 1:

a Especially, were groups comparable at baseline in key prognostic variables (e.g. age, vaccination status)?

b Especially, when some participants in one group but not the other are included in (or removed from) the denominator or differently followed up when outcomes are assessed.

c Especially, when people have received the intervention but have been classified as not having received it and vice versa.

d Especially, when one group receives additional measures to the intervention being assessed (e.g. advice or more frequent follow-up checks), or when the intended intervention is for some reason not delivered (e.g. due to lack of adherence).

e Especially, attrition (losses to follow-up) or missed appointments. Reasons for missing data must be explored.

f Especially, when the outcome of interest is misclassified (e.g. as not having developed the disease when the person did).

g Especially, when authors selectively reported those associations that turned out to be statistically significant.

While the above tools remain popular and are considered pragmatic and easy to use, they have been criticised for being vague, lacking validity, and scoring poorly on inter-rater reliability.^14^ ^,^ ^15^ In recent years, a group led by Cochrane epidemiologists and statisticians has worked on a new suite of tools that focus systematically on predefined domains of bias.

The Risk of Bias in Non-randomized Studies of Interventions (ROBINS-I) tool,^16^ for example, is used for cohort studies where one group was offered an intervention. It is designed to support not merely quality assessment but *causal inference*. The tool views each observational study as an attempt to emulate a hypothetical pragmatic RCT (referred to as the ‘target trial’) and assesses bias relative to this ideal using counterfactual reasoning (i.e. considering what *would* have happened had an ideal RCT been possible). A bias may be non-differential (same in each group), which will tend to bias findings towards null (i.e. attenuate any measured effect). If a bias is differential (occurring more in one group than in the other), it could bias findings either towards or away from the null. The ROBINS-I items, however, focus on the *presence* and estimated *magnitude* of bias rather than on its *direction*.

In the accompanying guidance to ROBINS-I, reviewers are exhorted not to simply ‘score’ a study but to undertake a detailed, reasoned assessment of its flaws.^16^ The tool’s developers also emphasise that both methods experts (in epidemiology and statistics) and topic experts are needed to apply the tool to a particular research question, though they warn that topic experts may be swayed by subjective impressions, prior assumptions, or conflicts of interest and should not be allowed to dominate deliberations.

Though comprehensive, ROBINS-I has been criticised for being time-consuming to complete^17^ and unreliable in the hands of reviewers who are not methodological experts, leading to a tendency to underestimate key bias domains and convey a false sense of certainty.^18^ A modified tool (ROBINS-I v2), which includes more detailed algorithmic guidance in an attempt to overcome these problems, was released in late 2024.^19^

ROBINS-I is promoted by Cochrane as a next-generation tool on account of its comprehensive coverage of theoretical domains of bias. JBI methodologists have recently mapped the criteria in their critical appraisal checklists to key bias domains (e.g. ‘bias relating to temporal precedence’, ‘bias relating to administration of intervention/ exposure’) while retaining the original wording of items.^15^ Like Cochrane, they have also lengthened the guidance and introduced a semi-quantitative (rather than yes/no) scoring system.

The ROBINS-E (‘E’ being ‘Exposure’) tool was developed for observational studies of exposures (including environmental, occupational, and behavioural factors), where RCTs are acknowledged to be less feasible.^9^ ^,^ ^20^ ROBINS-E shares many of the core features of ROBINS-I, including the ‘target trial’ analogy, a seven-domain structure for assessing bias, and a semi-quantitative scale (low, moderate, serious, critical) for those assessments. Again, accompanying guidance encourages reviewers to eschew technocratic scoring and engage with the study’s detail—a feature that makes the tool time-consuming to use and critically reliant on the reviewer’s expertise. The ROBINS-E tool has been criticised for omitting some key sources of bias in exposure studies, failing to discriminate studies with a single risk of bias from those with multiple risks of bias, failing to distinguish confounders from co-exposures, and providing limited insight into whether confounders will bias study outcomes.^9^ We address additional debates about this tool in the Discussion section.

The ROBINS tools (and, less ideally, other quality assessment tools) can feed into the Risk of Bias domain in the GRADE process, designed primarily to assess the certainty of evidence when developing guidelines. As Table 1 shows, GRADE also includes additional considerations. Primary studies may be at low risk of bias, but the body of evidence on a topic may be imprecise (e.g. wide confidence intervals), inconsistent (i.e. show unexplained heterogeneity), or less relevant to a particular setting (i.e. have low external validity). According to GRADE guidance, the level of certainty of a body of evidence may be upgraded if the effect size is large, if a dose–response relationship is demonstrated, or if the direction of residual confounding would be towards null.

While there is broad consensus that the assessment of study quality (including but not limited to risk of bias) is mission-critical in evidence synthesis of observational studies, there are inherent trade-offs in the various tools on offer, notably whether to favour succinctness and simplicity or depth and comprehensiveness. The emergence of increasingly detailed guidance on how tools should be used raises questions around the extent to which reviewers read and take note of such guidance (and what might happen if they do not). However rigorous a tool’s development, its use still depends on subjective human judgments.

### 1.4 Aims and research questions

Our aim was, using mask efficacy as an example, to examine the extent to which orthodox evidence synthesis methods such as ‘PRISMA-compliant’ systematic review and meta-analysis are fit for purpose for assessing and combining complex evidence (i.e. extensive, heterogeneous, and with complex causal pathways). We focussed primarily but not exclusively on observational evidence because this is where review methodology is most contested.

Our research questions were:

How did reviewers of complex evidence on mask efficacy produce a manageable dataset from a large number of potentially relevant studies? If they rejected all observational studies outright, how was this decision justified?

How did reviewers deal with heterogeneity (e.g. in terms of study design, settings, populations, mask types, diseases, and primary outcomes) of primary evidence on mask efficacy? If meta-analysis was used, how were decisions made about ‘lumping’ or ‘splitting’ primary studies?

How did reviewers assess study quality (variously, overall study quality, risk of bias, and certainty of evidence)? To what extent were quality assessment tools used skilfully and judiciously? To what extent were quality assessments consistent across different review teams?

What general lessons can be learnt from this example about synthesising complex evidence?

## 2 Methods

### 2.1 Description of study and dataset

This is a sub-study of a wider programme of work funded by a UKRI programme for interdisciplinary research. We are using the efficacy of face masks and mask mandates in reducing transmission of respiratory disease as an example through which to develop new, interdisciplinary methods of evidence synthesis. Full methods, including detailed search strategies, are described in a separate protocol paper.^1^ An interdisciplinary advisory board provides wider academic expertise and external scrutiny. As desk research, it was deemed not to require research ethics approval.

To briefly summarise our search strategy for identifying publications on mask efficacy, we supplemented keyword searches across multiple databases (PubMed, EMBASE, Social Science Citation Index) with additional methods, including authors’ prior knowledge of the topic, contacting topic experts, mining previous systematic reviews and primary studies, and forward-tracking selected studies in Google Scholar. Electronic searches on this complex topic proved neither sensitive nor specific, but the combination of methods allowed us to iteratively build a database containing dozens of previous systematic reviews and hundreds of primary studies up to September 2025. No language restriction was applied (though in practice most papers were in English); non-English language papers were appraised by multilingual academic colleagues with advanced training in systematic review methods.

We collated studies described by their authors as systematic reviews or meta-analyses (including ‘narrative systematic reviews’ but excluding narrative reviews without a formal search strategy or methodology). We downloaded the full papers and also all supplementary materials. We contacted authors if material referred to in the paper (e.g. item scores on quality instruments) was unavailable online. We created a spreadsheet summarising their country of origin, authors’ backgrounds, review focus, selection criteria for primary studies, methods (especially which quality assessment tools were used), key findings, and strengths and limitations. We also summarised all identified primary studies on a separate spreadsheet.

For the analyses presented in this article, we used our dataset of systematic reviews and considered how they had dealt with primary studies, especially observational designs (i.e. non-randomised ones, including case–control, cohort, cross-sectional, and ecological). All assessments were undertaken independently by two authors (TG and SR), with differences resolved by discussion. Where there was residual disagreement, the assessment was categorised as ‘uncertain’.

### 2.2 Analysis of systematic reviews of mask efficacy

We examined each of the 66 systematic reviews of mask efficacy for their funding source and author expertise. We classified an author as having methodological expertise if they had a degree in epidemiology, medical statistics, evidence-based medicine or similar discipline, or a clear track record of a substantive role in a previous systematic review. We classified them as having topic expertise if they had played a substantive role in non-laboratory empirical research studies of mask efficacy or infectious diseases more generally. This approach probably overestimated expertise, since we did not take account the quality of the authors’ previous empirical research.

We looked at the reasons given for *not* including observational studies if the authors had made this choice. We identified which primary observational studies had been covered in the remaining reviews and how decisions about eligibility, study quality, and aggregation (e.g. whether to do a meta-analysis) had been made. We selected a maximum-variety sample of five ‘tracker’ primary studies that had been included in at least five systematic reviews in our sample. These comprised: two studies of healthcare workers in the 2003 SARS outbreak (one unmatched case–control from Hong Kong^21^ and one retrospective cohort from Canada^22^); two studies of COVID-19 in community settings (one matched case–control from Thailand^23^ and one outbreak investigation [retrospective cohort] from China^24^), and one unmatched case–control study of influenza transmission on a flight from the USA.^25^ These were selected to provide a range of countries, settings, study designs, and contexts, and also to have been undertaken early enough to have featured in several systematic reviews.

For each tracker study, we identified all the systematic reviews that had included it. We critically examined how each systematic review had assessed the study, including whether and how it was included in meta-analyses, whether and how study quality tools were used, and any interpretive commentary or justification for scores awarded. We compared these approaches across reviews. This exercise was undertaken independently by two reviewers (TG and SR), and differences were resolved by discussion.

## 3 Results

### 3.1 Description of the dataset

Our search produced a dataset of 66 systematic reviews plus approximately 300 primary studies on mask efficacy, comprising 26 RCTs, 129 observational (cohort, case–control, and cross-sectional) studies and 145 ecological or modelling studies. Of these studies, 100 were in healthcare; most of the remainder were in the community; and two considered both. An additional literature on laboratory experiments on mask efficacy was outside of our scope. A table presenting descriptive data for the systematic reviews is available in Supplementary Material 1. Further details of the primary studies are available from the authors.

Of the 66 systematic reviews, 59 had been published since 2020. Forty reported no funding at all, and another four acknowledged only a personal salary for one author (though it is possible that even when no funding was acknowledged, the work may have been undertaken in paid time). The other 22 reviews had received public funding (government or WHO commissions, or research calls). Of the 66 reviews, 39 included authors with demonstrable expertise in evidence synthesis (e.g. an academic appointment in epidemiology); in a further nine reviews, at least one author may have had some training and experience in review methods. Ten reviews included an author with demonstrable expertise in researching the efficacy of masks in infectious disease outbreaks; in another seven reviews, one or more authors may have had some expertise in this topic.

Of these systematic reviews, 19 were restricted to community settings; 20 looked only at healthcare settings; and 27 considered both. Sixteen excluded observational evidence. Of the remaining 50, 24 included at least one statistical meta-analysis of observational studies and the other 26 presented data from observational studies in disaggregated form (e.g. in tables). Most reviews addressed all respiratory infections or respiratory viral infections; two were restricted to influenza and 13 to COVID-19.

Of the 66 reviews, only seven covered any ecological or dynamic modelling studies, and in only two were these designs the main focus of the review. This is a major limitation of the body of secondary literature as a whole, since infectious disease outbreaks show complex geographical and temporal dynamics and masking will have different (and changing) efficacy depending on the nature and phase of the outbreak.^26^ ^,^ ^27^ The strengths and limitations of ecological designs are beyond the scope of this article, but it is worth noting that reviews that cover only ‘static’ designs will provide, at best, a static picture of mask efficacy.

Overall, 37 of the 66 systematic reviews concluded that masks reduced the transmission of respiratory infections or that respirators (e.g. N95s) were more effective than medical masks in this regard. The remaining 29 concluded either that the evidence supported lack of efficacy or that definitive evidence either way was lacking.

### 3.2 How reviewers produced a manageable dataset

When (as with mask efficacy) the number of primary studies on a topic runs into hundreds, reviewers must either limit their focus or prioritise a subsample of studies for analysis. One commonly used strategy by reviewers was to reject observational evidence altogether—which in this dataset would reduce the number of primary studies by around 90%. Of the 16 reviews that excluded all observational evidence, 14 gave no reasons; one acknowledged the existence of an extensive body of observational evidence but said that this was likely to ‘introduce noise into findings’ (p. 2),^28^ and one considered observational evidence unnecessary because there were ‘sufficient’ RCTs to answer the review question (p. 10).^2^

Most review teams that chose to include observational evidence restricted the focus in some way—for example, by looking only at certain study designs (e.g. case–control studies), only at protection of healthcare workers, only at a particular disease, or only at special settings (e.g. pilgrims attending mass religious gatherings). This way, most reviews covered a manageable dataset of fewer than 40 primary studies. A few, however, retained a broad focus (and indeed, some chose to cover all non-pharmaceutical interventions, including masks, hand hygiene, physical distancing, and stay-at-home orders, in a single review). These authors faced a primary dataset that was potentially overwhelming, requiring them to make trade-offs between breadth and depth.

### 3.3 Dealing with heterogeneity

Even with explicit restrictions on study eligibility, most review teams still found themselves grappling with a sample that was highly heterogeneous and difficult to tame. Primary studies had addressed different research questions (e.g. source control versus wearer protection) in different diseases (influenza, SARS, MERS, COVID-19, bacterial superinfection), populations (e.g. healthcare workers, pilgrims attending mass gatherings, lay public), and settings (e.g. high risk such as infectious disease wards or households with an infected family member; or low risk such as communities where background prevalence of the disease was low). They had used different study designs (e.g. case–control, cohort, RCT), interventions (cloth masks, medical masks, respirators, with varying instructions for when and how to wear them), type of outcome (population-level epidemiological [e.g. number of confirmed cases], individual-level epidemiological [e.g. date of symptom onset], individual-level behavioural [e.g. self-reported adherence]), specific outcome measures (e.g. symptoms, clinical assessment score, laboratory test, rapid antigen test), and measures of effect size (secondary attack rate, RR, OR, RD).

While tables of study characteristics sometimes conveyed a broad sense of this heterogeneity, they rarely fully made sense of it. Absent from the reviews was an overall sense of *why* different studies had approached mask efficacy in widely differing ways and the contribution made by different study designs and settings to the overall picture. Indeed, with few exceptions and notwithstanding ‘grand means’ generated by meta-analysis, there *was* no overall picture.

The number of primary studies included in meta-analyses ranged from one to several hundreds (see Supplementary Material 1). Primary studies were lumped and split in different ways, including by study design (e.g. cohort studies analysed separately from case–control), details of intervention (e.g. whether N95s were worn continuously or intermittently in healthcare settings), or primary outcome (e.g. influenza-like illness, clinical respiratory illness, serologically confirmed clinical symptoms, or PCR test). Twelve reviews combined different study designs in a single meta-analysis. One of these split a large dataset in multiple ways: healthcare versus community, Asian versus non-Asian (a proxy for whether attitudes to masks were positive), and virus type (influenza, SARS, or COVID-19).^29^ Another combined household transmission studies (in which a symptomatic family member was the index case) with healthcare settings on the grounds that both are high-risk settings for disease transmission.^30^

As shown in Table S1 in the Supplementary Material 2, our five tracker studies^21^ ^–^ ^25^ were included in 11, 17, 15, 4, and 4 separate meta-analyses, respectively. In each case, the study was combined with a different selection of other studies, and (unsurprisingly) the analysis produced a different OR for mask efficacy. In all but one case, however, the meta-analysis demonstrated a significant effect of masks compared with no masks and for N95s (or other respirators) compared with medical masks.

Almost all meta-analyses of observational evidence in our dataset included every study deemed eligible for inclusion by the authors, whatever the risk-of-bias score assigned. Thus, studies at critical risk of bias were combined statistically with those at low risk of bias, with no adjustment. One review (Chu et al.^31^) omitted primary studies from the meta-analysis if they had not adjusted for confounders. Another review (Yanni Li et al.^32^) offered a sensitivity analysis in which they used raw data from primary studies to adjust for confounders when the authors of the primary studies had not done so; these adjustments did not change the overall effect size estimate. As noted by Paul et al., stringent omission of unadjusted observational studies can produce an ‘empty’ review since many such studies do not adequately adjust for confounders and raw data may not be forthcoming from authors.^33^

### 3.4 Assessment of study quality

The 50 systematic reviews in our sample that included observational studies used various instruments to assess their quality. These comprised the NOS,^13^ JBI critical appraisal checklists for cohort and case–control studies,^12^ US Preventive Services Task Force checklist for observational studies,^34^ CASP checklist for cohort and case–control studies,^11^ STROBE reporting guidelines for observational studies,^35^ National Heart, Lung and Blood Institute (NHLBI) quality assessment checklist,^36^ ROBINS-I,^16^ and ACROBAT-NSRI (an early version of ROBINS-I developed by Cochrane).^37^

Two reviews developed bespoke risk-of-bias tools for their datasets (one by adapting NOS^38^ and one by adapting a previous bespoke tool for ecological studies^39^). No review used ROBINS-E, which was perhaps surprising (while ‘masking’ is an intervention, ‘being unmasked’ in an infectious environment was sometimes described as an exposure). Nineteen reviews either did not use a quality tool for observational studies or (in three cases) provided no results for the tool they did use, though one out of the three supplied these data when we requested them.

**Figure 1:**
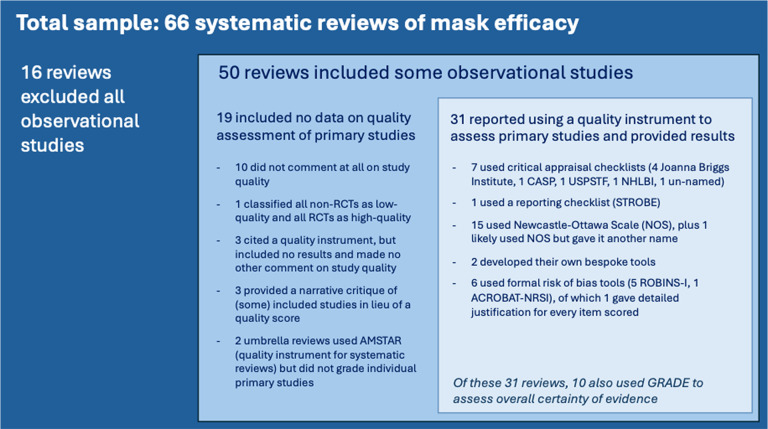
*How 66 systematic reviews addressed the quality of primary observational studies of mask efficacy*. Figure 1 Long description.

Figure 1 shows how the 66 systematic reviews dealt with observational evidence. This highly varied treatment of observational evidence contrasts with the way reviewers assessed risk of bias in RCTs: 28 out of the 33 reviews that covered RCTs used the Cochrane risk-of-bias tools.

Pursuing our five selected tracker studies^21^ ^–^ ^25^ through all the systematic reviews that had included them revealed widely differing judgments about their quality. Details of this analysis are provided in the Supplementary Material 2 (see Tables S2–S12). We illustrate our findings here using a community-based matched case–control study from Thailand undertaken during the COVID-19 pandemic by Doung-Ngern et al.^23^ This study was covered by 11 of the 66 systematic reviews.^32^ ^,^ ^40^ ^–^ ^49^ Of these, four cited the NOS,^32^ ^,^ ^43^ ^,^ ^45^ ^,^ ^47^ though one gave no data and the remainder only gave an overall score (8/9, 9/9 and 9/9, i.e. all ‘high quality’). Two used the JBI checklist for case–control studies; one scored every domain as ‘yes’ (low risk of bias), producing an overall score of 12, while the other scored only four domains as ‘yes’, which would produce a score of 4.^48^ ^,^ ^49^ One used the US Preventive Services Taskforce checklist and assessed overall study quality as ‘poor’.^42^ Three reviews used the ROBINS-I; their assessments, which produced overall scores of ‘serious’, ‘serious’, and ‘moderate’ risk of bias, are reproduced in Table 2.^40^ ^,^ ^44^ ^,^ ^46^ One review did not use a risk-of-bias score.^41^ Only one review^44^ provided a discussion of *what the main biases were* and estimated the direction of these biases.

**Table 2:** Scores awarded by systematic reviews to the Doung-Ngern study using robins-I Table 2 Long description.

	Floriano^46^	Kim^44^	Talic^40^
Confounding	Low	Low	Low
Selection of participants	Moderate	Low	Serious
Classification of interventions	Low	Low	Moderate
Deviations from intended interventions	Moderate	Moderate	Serious
Missing data	Serious	Low	Moderate
Measurement of outcomes	Low	Low	Moderate
Selection of reported results	Low	Low	Moderate
Overall assessment	Serious	Moderate	Serious
Narrative	None	See text	See text

Similar analyses on four other tracker studies^21^ ^,^ ^22^ ^,^ ^24^ ^,^ ^25^ are available in Supplementary Material 2. They confirm wide variation in assigned scores (e.g. scores on a single item on the NOS ranged from 2/9 to 7/9; on one ROBINS-I subdomain, scores ranged from ‘low risk of bias’ to ‘critical risk of bias’). We consider the implications of this variation in the Discussion section.

### 3.5 Limited narrative

A striking feature of most reviews in our dataset was the limited amount of information about primary studies that was provided outside the structured tables of predefined data fields. Particularly in reviews covering large numbers of studies, information from primary studies was compressed into lengthy tables (usually supplied only as online supplements to the main paper). Accompanying narratives to *explain* these structured data summaries and justify the scores awarded were absent or extremely brief, even in the supplements.

Only one review in our sample (Kim et al.^44^) set out a detailed narrative justification for every risk-of-bias judgment for every study (as recommended by the ROBINS-I authors^16^). A few reviews (see, for example, Talic et al.^40^ and Chen et al.^43^) included brief comments on limitations in their tables of included studies. In addition, three reviews which did not use a formal score or checklist provided a narrative critique of at least some observational studies in the text of the paper.^41^ ^,^ ^50^ ^,^ ^51^ But all these examples were the exception. In most reviews, the risk of bias or other quality assessment was expressed *only* as a numerical score or ordinal category (e.g. ‘moderate’).

Yet a close read of the Doung-Ngern case–control study,^23^ for example, revealed many distinctive features that were not captured in any of the systematic reviews. For example, this study was undertaken at a very early stage in the COVID-19 pandemic when community transmission was only just beginning, meaning that contamination from other community contacts was highly unlikely. The research team asked detailed questions about when and how masks were worn when in the vicinity of the index case, allowing them to estimate fidelity of the intervention and test the hypothesis that to be effective, masking must be continuous. This hypothesis was supported in an important negative finding: people who wore masks only *some* of the time when in contact with an index case were no less likely to develop COVID-19 than those who did not mask at all, whereas those who *continuously* wore masks were significantly less likely to do so. This finding aligns with background evidence that respiratory diseases are airborne, so infectious particles can spread to fill an indoor space and may remain viable in the air for minutes or even hours after being generated in a cough, sneeze, or exhaled air.^52^ Additionally, the study provided early evidence to challenge the ‘risk compensation’ hypothesis that was popular at the time: people who wore masks were significantly *more* likely to wash their hands regularly, practise social distancing, and spend *less* time in close contact with others than those who did not.

The explanations in Kim et al.’s comparative effectiveness systematic review of respirators, medical masks, and cloth masks were important.^44^ An example is missing data, a well-recognised source of bias in case–control studies. In the Doung-Ngern study, data on whether the *index case* had worn a mask was missing in 27% of people interviewed. When justifying their score of ‘low risk of bias’ for missing data in this study, Kim et al. stated: ‘Missing values for mask wearing [by index cases] not included in analyses. For other variables, missing values were [assumed to be] random and were imputed by chained equations.’ This brief narrative, along with details of the imputed datasets, illuminates a *judgment* about the extent to which missing data influenced the trustworthiness of the findings, given the study’s authors’ own decisions to exclude them or their attempts to correct for them. It explains why the score they awarded differs widely from that given by Floriano et al. using the same tool.^46^ The latter authors give no narrative and appear to have treated the item as a simple yes/no question of whether some data were missing. Given the variability in scores across reviews, this absence of narrative justification in all but a handful of reviews is concerning.

Few systematic reviews in our sample included any reasoning about the likely *direction* of biases identified. In the Doung-Ngern case–control study, for example, a key bias is that only 89% of the controls were tested for SARS-CoV-2. The authors reasoned that missed individuals were likely to be true negatives because they had been in less close contact with the index case and for less time (at a time when community prevalence of the disease was very low), but as one or two systematic reviews pointed out, *some* of these individuals could have been misclassified. However, such misclassification would tend to bias findings towards null, so a statistically highly significant result *despite* these missing data is unlikely to be spurious. We conducted a sensitivity analysis which showed that even if half of the untested controls were actually positive (perhaps asymptomatic) COVID-19 cases, the crude OR would change only from 0.72 to 0.79 and the adjusted OR from 0.23 to 0.26.

We detected a marked shift in style and tone from the earliest systematic review in our dataset to the latest. Cowling et al. provided a detailed explanatory account of the 11 studies published at the time, stating that a meta-analysis was inappropriate because of heterogeneity.^51^ In contrast, Chen et al., whose sub-study on mask efficacy is part of a wider review on the impact of adherence to masking, listed no fewer than 448 primary studies representing 654 datasets in a single supplementary table and offered various meta-analyses of these (though not all studies addressed mask efficacy); no primary study was described or critiqued in the main paper.^53^ While this was an extreme example, almost all reviews published since 2020 offered extensive structured data but lacked explanatory detail.

In most cases, the conclusions of these systematic reviews were bland, unnuanced, and non-definitive (e.g. ‘There is limited, low certainty direct evidence that wearing face masks reduces the risk of transmission of SARS-CoV-2 in community settings. Further high-quality studies are required to confirm these findings.’ (p. 2)^49^). Such statements, while not incorrect, suggest that many review teams had either been unable to identify high-quality *existing* primary studies (especially observational ones) or were not confident to draw firm conclusions from these studies.

## 4 Discussion

### 4.1 Summary of principal findings

This study of evidence synthesis methods for mask efficacy studies has revealed some findings that are concerning but perhaps not surprising given that most were undertaken during the COVID-19 pandemic—a time when research on pandemic-related topics expanded rapidly, pressure to produce outputs was high, and quality control was variable. Hundreds of primary studies have addressed the topic, but fewer than 10% of these were RCTs; the remainder were a highly heterogeneous collection of observational, ecological, modelling, and other designs, presenting challenges for review teams. Most systematic reviews of these studies were done without dedicated funding and hence without a budget for bringing in specialist expertise or protected time for the close reading and deliberation needed to make judgments about complex evidence.

Review teams handled observational evidence in a variety of ways. In a few cases, reviewers combined methodological and topic expertise and applied the newer, more extensively validated risk-of-bias tools appropriately to produce a synthesis that was detailed, nuanced, and authoritative. Many review teams, however, appeared to lack methodological expertise, topic expertise, or both; they tended to use older, shorter, and less robust risk-of-bias tools. Furthermore, such tools were sometimes used inconsistently and superficially, producing unreliable scores offered with no explanation or justification. Various moves by reviewers—excluding observational evidence altogether, assessing risk of bias but not its direction or importance in a particular study and context, omitting distinguishing details of primary studies, and presenting meta-analyses that combined studies of different designs or included those at critical risk of bias—served to obscure important aspects of heterogeneity, resulting in bland, non-definitive, and conflicting summary statements. Interpretive syntheses to explain the nuances across heterogeneous primary evidence were largely absent.

These important limitations of many systematic reviews of mask efficacy were evident *despite* evidence (in most cases) of authors’ diligent efforts to produce a ‘PRISMA-compliant’ review characterised by a thorough search, systematic assessment of primary studies, ‘objective’ risk-of-bias scoring, and meticulous tabulation of such scores.

### 4.2 Implications

While the example of systematic reviews of mask efficacy undertaken mostly during the COVID-19 pandemic may not be representative of systematic reviews more generally, we believe our findings justify four key conclusions. First, more attention needs to be given to the training and oversight of authors who take on systematic reviews of complex evidence, especially in the selection and use of risk-of-bias tools. Second, both methods experts *and* topic experts appear to be needed to optimise the operationalisation and application of such tools to make judgments. Third, reviewers should publish (or make available) not only the outputs of their assessments of primary studies but sufficient rationale to allow readers to understand, and be able to agree or disagree with, *how* the tools were operationalised to the topic and the main judgments reached. And finally, reviews would be more informative if they indicated, for each study, the direction of each bias (towards null, away from null, or either way) as well, simply identifying whether it is present.

### 4.3 Comparison with previous literature

Our findings illustrate how the commendable efforts by methodologists to develop robust and reliable tools and guidance for synthesising observational evidence^10^ ^,^ ^16^ ^,^ ^19^ ^,^ ^54^ have, at least in some public health topics, been offset by three things: the vast expansion in the volume and heterogeneity of observational evidence;^7^ ^,^ ^55^ the growing tendency of inexperienced review teams to take on complex evidence synthesis projects;^18^ and (real or perceived) pressures to generate policy-relevant systematic reviews at pace during periods of crisis.^56^ While several high-quality systematic reviews existed, they were greatly outnumbered by lower-quality ones.

Our finding that there was no consistency in how review teams handled heterogeneity aligns with other studies in the literature. Published advice on this topic focusses almost exclusively on approaches to *statistical* heterogeneity.^6^ ^,^ ^57^ ^,^ ^58^ Mueller et al. summarised advice on synthesis of observational evidence from 93 previous articles in their 2018 systematic review.^57^ These articles gave different advice on which criteria should be used to lump or split studies in meta-analyses, though most methodologists considered that analyses should be split by study design. Recommendations also differed on the value of quality scores (including risk-of-bias) when used across heterogeneous datasets. While some scholars viewed these tools as an essential component of a good review, others were concerned that expressing quality judgments in terms of a *summary* score was problematic since a) observational studies are subject to many different biases, b) these different biases may be additive, multiplicative, or cancel out, and c) use of such scores can (in the words of one quoted commentator^59^) ‘seriously obscure heterogeneity sources’.^57^

While there is a move among review methodologists to recommend against meta-analysis when primary studies are highly heterogeneous (see, for example, the SWiM [Synthesis Without Meta-analysis] reporting guidance^60^), our own finding that none of the 66 reviews used SWiM suggests that this advice has not reached most of the people undertaking such reviews.

Ours is not the first study to demonstrate that risk-of-bias assessment can be particularly unreliable in observational studies. Some controlled experiments designed to test the performance of such tools showed high inter-rater reliability when used by skilled reviewers.^61^ ^,^ ^62^ But other similar experiments showed moderate to poor inter-rater reliability.^14^ ^,^ ^63^ ^,^ ^64^ Inter-rater reliability improved substantially after the review team received training^65^ and when reviewers spent more time on each assessment.^66^ The more detailed tools such as ROBINS-I were experienced as ‘difficult and demanding’ even by skilled reviewers,^62^ and papers have been written on ‘assessor [or evaluator] burden’.^64^ ^,^ ^66^

Our study differed from these publications in that we did not set out to formally test risk-of-bias tools by comparing how trained reviewers scored a pre-selected sample of papers. Rather, we looked at the published results of systematic reviews undertaken ‘in the wild’ to see how the tools had fared in the hands of review teams, many of whom were under-resourced, lacked methodological or topic expertise, and were attempting to deal with complex datasets of primary evidence.

Our findings align with those of two other overviews of systematic reviews. Mei et al. showed that most of their sample of 220 systematic reviews had assessed risk of bias in non-randomised studies of interventions using the NOS or other first-generation tools, while very few used ROBINS-I.^55^ Igelström et al. looked only at ROBINS-I; they found that in 124 systematic reviews across a wide range of topic areas, misapplication of this tool was common.^18^ In particular, 89% of these reviews included no explanation or justification for scores awarded and 20% included studies classed as ‘critical risk of bias’ in meta-analyses. Whereas Mei et al. and Igelström et al. considered the ‘in the wild’ use of risk-of-bias tools across multiple topics, our own study considered the use of such tools (or no tools) by a sample of reviews on a single topic.

### 4.4 Strengths and limitations of this study

The key strength of this study is our novel methodology of close reading of an entire dataset of all available systematic reviews on a complex and contested topic, including tracking a sample of primary studies through all reviews that included them to reveal variation in the depth of analysis and final scores awarded. We approached our sample of reviews not primarily with data extraction in mind but with a view to understanding why review teams ‘in the wild’ made the choices they did and how they selected and used tools for study quality. As such, our study complements previous work on the usability and reliability of risk-of-bias tools conducted in experimentally controlled settings. A limitation of our method is that we were unable to generate formal inter-rater reliability scores, but (unsurprisingly) we were able to demonstrate wide and frequent discrepancies across reviews and unpack, item by item, not just the range of scores awarded but *why* review teams differed so widely.

### 4.5 Philosophical reflections on risk-of-bias tools

Our discovery that systematic reviews of mask efficacy can produce unreliable findings despite the use of risk-of-bias (and other quality assessment) tools—and indeed, that in non-expert hands, these tools may *reduce* the reliability of the findings—raises philosophical questions about the place of such tools in evidence synthesis. These questions relate to (among other things) the nature of the knowledge that can be captured in the tool; the nature of the knowledge required to use the tool; and the nature, magnitude, and direction of the biases that the tool is intended to evaluate. Observational evidence brings these questions to the fore, since the biases are multiple and complex and play out differently (and in different directions) in different study designs.

Some of these philosophical questions have been touched upon in a recent public disagreement among tool developers. Steenland et al., who were originally part of the development group for ROBINS-E, explained in an open letter why they withdrew their names from all publications related to it.^67^ Their concerns include that the ‘target trial’ analogy is inappropriate for exposures (they believe) as the mechanisms of exposure and disease may not align with the intervention model of an RCT; the structure of the bias domains creates high potential for unnecessary exclusion of high-quality studies; the tool places insufficient emphasis on how triangulation using other kinds of evidence (see, e.g., Lawlor et al.^68^) can help mitigate biases in exposure studies; it does not consider the direction of bias or the extent to which bias in one direction might balance out a different bias in the other direction; and there is no domain for either the quality of statistical methods or conflict of interest.

These points echo those made in an earlier paper by Arroyave et al.,^69^ who argue that risk of bias in observational studies should be assessed either using a tool that compares each study with a hypothetical ideal *observational* study (as occurs in some toxicology syntheses) or by constructing a bespoke tool for each topic using precise criteria for each risk-of-bias domain relevant to the exposure–outcome relationship under study.

In a response to Steenland et al.’s open letter,^67^ the remaining ROBINS-E authors expressed concern that their detractors’ proposed alternative approach, based substantially on the judgments of topic experts rather than methods experts, is unsystematic and subjective (and hence, unreplicable) and that while triangulation is a promising approach for complementing the use of ROBINS-E, it currently ‘lacks a formal framework’ (p. 1).^70^

This debate reflects a polarisation that can be traced to foundational assumptions about the nature of knowledge for evidence synthesis. Orthodox review methodologists assume that, notwithstanding the limitations of any particular tool, it is theoretically possible to develop generic tools that will, in the right hands, produce valid and replicable estimates of risk of bias for each study design. These scholars consider that the expertise needed is predominantly methodological (i.e. related to the methodology of evidence synthesis) and that while topic experts may need to be consulted, they should not be making the key methodological decisions since, as leading Cochrane methodologists once claimed, ‘[topic] expert advice is often unreliable’ and such experts ‘may have personal prejudices and idiosyncrasies’.^71^

Those who reject the assumed theoretical benefits of a rigorously developed risk-of-bias tool do so on both practical and philosophical grounds. Practically, Savitz et al. cautioned that whatever caveats are added by a tool’s authors, in reality such tools are almost always applied ‘mechanistically’, prioritising repeatability of scoring over depth of analysis and paying scant or no attention to whether a particular bias is actually meaningful or important in any particular topic area.^72^ Philosophically, the grounds for rejection can be explained in terms of Wittgenstein’s rule-following paradox, namely that ‘no course of action could be determined by a rule, because any course of action can be made out to accord with the rule’ (*Philosophical Investigations, §201*).^73^ That rules are not self-interpreting does not, in fact, mean that all rules should be abandoned (as a reviewer of an earlier draft of this article pointed out, this would effectively kill the science of systematic review). It means that however rigorous the rule and however detailed the instructions are for applying it, there will always be an element of interpretation and judgment involved, and novel cases will arise that are not covered by existing instructions. As an early set of guidance from the US Association for Healthcare Research and Quality put it, ‘Using transparent rules does not remove the subjectivity inherent in assigning the risk of bias category’ (p. 19).^74^ Accordingly, we suggest that the task of applying a rule in any given case requires not merely review methodology knowledge (e.g. what domains to cover about risk of bias in general) but topic-based expert judgment (what domains to cover, and how these should be operationalised, *in relation to a specific scientific topic*)—and perhaps most importantly, *deliberation* between methods experts and topic experts on how to fit the tools to the topic.

To accurately evaluate study quality, generic biases (e.g. ‘misclassification of exposure’) must be translated into topic-specific ones (e.g. classifying whether someone was adequately masked when in a potentially infectious setting). To date, this crucial act of translation has received little attention from either tool developers or philosophers of science. It requires detailed topic knowledge—for example, knowledge that because respiratory diseases are airborne, indoor crowded settings are of particularly high risk and masking must be continuous while indoors (rather than restricted to one or two metres from suspected infectious people). Philosophically, such knowledge is classified as *indirect* or *background* knowledge—that is, knowledge that relates to a hypothesis via a long chain of reasoning using auxiliary claims.^75^ Our findings that many systematic reviews a) do not include topic experts and b) provide no detail on how generic items were operationalised suggest that much further work is needed to optimise this important aspect of evidence synthesis.

In his PhD thesis on the philosophy of evidence synthesis (using mask efficacy as one example), Begun observes that in systems with high causal complexity, individual pieces of evidence can provide only noisy and indirect access to underlying phenomena. In such cases, hypothesis-testing requires not merely consideration of direct evidence (i.e. evidence that relates to the hypothesis through a short chain of reasoning), but also ‘background knowledge [which] typically allows researchers to reliably judge when the hypothesis space is exhaustive’ (p 44). In other words, Begun views topic expertise as essential to ensure that a full and detailed explanatory account of the evidence is produced. While orthodox systematic review (PRISMA) methodology does not direct reviewers to ignore background knowledge, it does not explicitly encourage them to map the hypothesis space as a preliminary step to seeking empirical studies, nor does it say *how* methods experts and topic experts should work together on reviews.

## 5 Conclusion

The evaluation of the role of face masks in preventing respiratory infections is a paradigm case in synthesising complex (i.e. extensive, heterogeneous, technically specialised, conflicting, contested) evidence. Through a close analysis of 66 existing systematic reviews, we have shown how, even when using formal tools, review teams find such evidence hard to assess and summarise, and they may therefore reach divergent conclusions. We believe our findings raise important questions about how generic risk-of-bias tools are currently used, especially in situations where the primary evidence demands expert understanding and tailoring of risk-of-bias questions to topic. In an ongoing research study, we are seeking to develop novel review methods for complex evidence which take a systematic approach to a) deciding which studies (especially, which observational ones) to include; b) dealing with heterogeneity; c) selecting and operationalising risk-of-bias tools (including when and how to adapt these to topic); and d) combining the use of formal tools (to maximise replicability) with interpretive methods (to provide context and nuance). It is too early to say whether these proposed new methods will improve the validity and reproducibility of reviewer decisions, but that is our ultimate aim.

## Supporting information

10.1017/rsm.2026.10072.sm001 Greenhalgh et al. supplementary material 1 Greenhalgh et al. supplementary material

10.1017/rsm.2026.10072.sm002 Greenhalgh et al. supplementary material 2 Greenhalgh et al. supplementary material

## Data Availability

All data are in the public domain.

## Supplementary material

To view supplementary material for this article, please visit http://doi.org/10.1017/rsm.2026.10072.

## Long descriptions

### Table 1 Long description

The table is organized into three columns: Described by the authors as, Questions or domains, and Scoring. It covers four major tools.

1. Joanna Briggs Institute checklist for aetiology and risk (2003). Described as a Critical appraisal checklist. It contains 10 questions focusing on group comparability, matching, identification criteria, exposure measurement validity, confounding factors, outcome assessment, and statistical analysis. Scoring is binary (yes, no, unclear, or not applicable) with an overall sum.

2. Newcastle-Ottawa scale for case-control studies (1999). Described as a Quality assessment tool. It features 8 items across three domains: Selection (case definition, representativeness, control selection), Comparability (confounders), and Exposure (ascertainment, follow-up). Scoring uses a star system up to 9 stars, categorized as low (0 to 3), medium (4 to 6), or high (7 to 9) quality.

3. R O B I N S dash I (2016). Described as a Risk of bias tool for causal inference. It assesses 7 domains: Confounding, Selection of participants, Classification of interventions, Deviations from intended interventions, Missing data, Measurement of outcomes, and Selection of reported results. Each domain is rated from low to critical, with the lowest-scoring domain determining the overall risk.

4. G R A D E criteria for non-randomised studies (2011). Described as a Guideline development framework. It evaluates 5 domains: Risk of bias, Inconsistency, Indirectness, Imprecision, and other considerations like dose-response. Certainty is graded as high, moderate, low, or very low.

Navigate back to Table 1.

### Figure 1 Long description

The diagram begins with a header stating a total sample of 66 systematic reviews of mask efficacy.

On the left, 16 reviews excluded all observational studies.

On the right, a large box contains 50 reviews that included some observational studies. This group is split into two columns.

The left column describes 19 reviews that included no data on quality assessment of primary studies. Bullet points specify

- 10 did not comment at all on study quality

- 1 classified all non-R C Ts as low-quality and all R C Ts as high-quality

- 3 cited a quality instrument but included no results and made no other comment on study quality

- 3 provided a narrative critique of some included studies in lieu of a quality score

- 2 umbrella reviews used AMSTAR but did not grade individual primary studies.

The right column describes 31 reviews that reported using a quality instrument to assess primary studies and provided results. Bullet points specify

- 7 used critical appraisal checklists including 4 Joanna Briggs Institute, 1 CASP, 1 U S P S T F, 1 N H L B I, and 1 un-named

- 1 used a reporting checklist S T R O B E

- 15 used Newcastle-Ottawa Scale N O S plus 1 likely used N O S but gave it another name

- 2 developed their own bespoke tools

- 6 used formal risk of bias tools including 5 R O B I N S-I and 1 ACROBAT-N R S I of which 1 gave detailed justification for every item scored.

A footer note states that of these 31 reviews, 10 also used GRADE to assess overall certainty of evidence.

Navigate back to Figure 1.

### Table 2 Long description

The table consists of four columns. The first column lists seven bias domains and an overall assessment. The subsequent three columns represent the systematic reviews by Floriano, Kim, and Talic.

* Domain 1. Confounding: Low for all three reviews.

* Domain 2. Selection of participants: Moderate for Floriano, Low for Kim, Serious for Talic.

* Domain 3. Classification of interventions: Low for Floriano, Low for Kim, Moderate for Talic.

* Domain 4. Deviations from intended interventions: Moderate for Floriano, Moderate for Kim, Serious for Talic.

* Domain 5. Missing data: Serious for Floriano, Low for Kim, Moderate for Talic.

* Domain 6. Measurement of outcomes: Low for Floriano, Low for Kim, Moderate for Talic.

* Domain 7. Selection of reported results: Low for Floriano, Low for Kim, Moderate for Talic.

* Overall assessment: Serious for Floriano, Moderate for Kim, Serious for Talic.

* Narrative: None for Floriano, See text for Kim, See text for Talic.

Navigate back to Table 2.
