# Supplementary material for: Synthesis challenges in complex evidence: A critical analysis of systematic reviews of face mask efficacy
Source: Res Synth Methods. 2026 Feb 6;17(4):714–33. doi: 10.1017/rsm.2026.10072 (PMC13311340; doi:10.1017/rsm.2026.10072)
Supplement: Greenhalgh et al. supplementary material 1 — Greenhalgh et al. supplementary material [file S1759287926100726sup001.docx]

# Appendix: Systematic reviews of mask efficacy (n = 66)

To identify systematic reviews of mask efficacy, we snowballed from reviews known to us and recommended by topic experts. We searched electronic databases including PubMed, Social Science Citation Index and Bodleian SOLO using key words (the full search strategy is given in the protocol paper (Greenhalgh et al., 2025)). We included reviews that covered mask efficacy in real-world settings (community and healthcare) but excluded those focusing solely on laboratory studies, cleaning or re-use of masks, powered air purifying respirators, or modelling hypothetical data. We include one additional review (#67) describing methods used in systematic reviews of mask efficacy.

Abbreviations:

HCW = healthcare worker, CASP = Critical Appraisal Skills Programme, SIGN = Scottish Intercollegiate Guidelines Network, CI = confidence interval, OR = odds ratio, RR = risk ratio, NPI = non pharmaceutical intervention, PPE = personal protective equipment

Unless otherwise stated, numbers in parentheses indicate the 95% confidence interval

Orange shading = review was restricted to RCTs (n = 16); 12 included a new meta-analysis of those trials and one included an ‘umbrella’ meta-analysis (of other meta-analyses)

Green shading = included observational studies with a new meta-analysis of such studies (n = 25)

Blue shading = included observational studies but no new meta-analysis of those studies (n = 25); one of these offered a new meta-analysis of RCTs

Pink shading = methods review (n = 1), not included in the dataset of 66 reviews

| Author / year / country | Scope | Meta-analysis of | | Studies included and ROB tool(s) used | Main findings | Comment (including evidence for mask efficacy or superiority of N95s Y/N/equivocal) |
| --- | --- | --- | --- | --- | --- | --- |
|  |  | RCTs? | Obser-vational studies? |  |  |  |
| 1. Abboah-Offei et al. (2021) India | Masking in prevention of respiratory infections (HCW and community) | N | N | Narrative review. 58 studies: 13 systematic reviews, 45 “quantitative studies” including “RCTs, retrospective cohort studies, case-control, cross-sectional, surveys, observational and descriptive studies”. No meta-analysis. No ROB tool. | Concludes that face mask use may prevent infection in the wearer and contribute to source control. | Equivocal. This review, while employing a systematic search strategy, is largely descriptive and uncritical of statements made in included papers. |
| 1. Aggarwal et al. (2020) India | Masks in community settings | Y | N | 8 RCTs in meta-analysis, no other studies. Cochrane ROB (no narrative justification fpr decisions). | No significant reduction in ILI either with masks alone (5 studies, pooled effect size: -0.17 [95% CI 0.43-0.10]; P = 0.23) or masks with handwash (6 studies, -0.09 [-0.58-0.40]; P = 0.71). | N. One trial omitted (because source control); others combined in a single meta-analysis. |
| 1. Alhajaji et al. (2024) | Masking to prevent resp infections in Hajj | N | Y | 10 observational studies all of Hajj pilgrims. 2 nested case-control, 8 prospective cohort. NOS. Meta-analysis (all combined). | No sig effect of masks. Sig lower inc of resp infections in masks “most of the time” v “some of the time”. | N. Weak primary studies; self-reports mostly (though these authors describe quality of primary studies overall as “good”. |
| 1. Alihsan et al. (2022) | Efficacy of face masks in preventing SARS-CoV-2, community + healthcare | N | N | 13 studies, all observational. | Non-standard analysis, they just added up the total number of mask-wearers and non-mask-wearers who caught COVID-19. “The probability of getting COVID-19 for mask wearers was 7% (97/1463, p=0.002), for non-mask wearers, probability was 52% (158/303, p=0.94).” | Y. Highly suspect analytic approach. Preprint. |
| 1. Alkhalaf et al. (2023) Saudi Arabia | Masks v respirators for prevention of HCW infection in dental settings | N | N | 5 studies included: 1 lab study, 3 systematic reviews (Bartozko 2020, de Araujo 2021, Samaranayake 2020), 1 network meta-analysis (Yin 2020). No new meta-analysis. No new analysis of RCTs. AMSTAR-2 and ROBIS tools for systematic reviews, no tool for primary study. | Raw count: 1 lab study and 1 systematic review showed non-inferiority of surgical masks; 1 review showed superiority of respirators if worn continuously; 1 found that better protection is achieved when source uses surgical masks than when recipient uses an N95 respirator; 1 concluded that surgical masks or N95 respirators alone do not provide full protection. | Y. ROB assessed using AMSTAR-2 and ROBIS for systematic reviews and Health Evidence tools. Raw data not analysed. |
| 1. Barasheed et al. (2016) Saudi Arabia | Masks for preventing infections in mass gatherings (community) | N | N | 2 RCTs and 23 observational studies (but only 13 produced a quantitative estimate of mask efficacy). No meta-analysis. No ROB tool used but pyramid of evidence used to rank study designs. | 2 negative RCTs, many positive observational studies but noted to have numerous flaws. Adherence to masking ranged from 0.02% to 93% (median ~50%). Sub-analysis suggested that adherence to masking reduced risk of respiratory infection. | N. Many included primary studies measured only subjective endpoints e.g. respiratory symptoms. |
| 1. Bartoszko et al. (2020) Canada | Masks and respirators in HCWs | Y | N | 4 RCTs in meta-analysis, no other studies. Cochrane ROB (no narrative justification). GRADE | Compared with N95 respirators; medical masks did not increase lab-confirmed viral respiratory infection (OR 1.06 [0.90-1.25]) or clinical respiratory illness (OR 1.49 [0.98-2.28]). | N. All RCTs combined in a single analysis |
| 1. Barycka et al. (2020) Poland | Efficacy of respirators in preventing transmission of airborne infections (HCW and community) | Y | N | 6 RCTs in meta-analysis, no other studies. Cochrane ROB (no narrative justification). | Pooled analysis showed that N95 respirators did  not reduce the risk of infection with respiratory viruses compared with medical/surgical masks (5.7% vs. 7.9%; RR = 1.12 [0.88–1.41]). | N. 3 observational studies (2 case-control, 1 cohort) were flagged but their findings were not described. |
| 1. Benkouiten et al. (2014) France | Masks and other measures in preventing respiratory infections in Hajj pilgrims | N | N | 28 studies, mostly surveys, correlating respiratory symptoms with whether pilgrims wore masks. No RCTs. No ROB tool used. | Primary studies highly contradictory, with some reporting large differences between masked and unmasked pilgrims in respiratory infections and others reporting no effect. | N. Authors comment that compliance is a major issue. Whereas pilgrims comply closely with hand washing, compliance with masking varies. |
| 1. Bin-Reza et al. (2012) UK | Masks in prevention of pandemic flu (HCW and community) | N | N | 8 RCTs (5 in community, 3 in HCWs, mostly in influenza), 9 retrospective observational (8 case-control, 1 cohort) studies, all in SARS. No ROB tool used. | Presents disaggregated results of individual trials but no meta-analysis. But gives raw count: 6 of 8 RCTs showed no evidence of efficacy; 8 of 9 retrospective observational studies found that mask and ⁄ or respirator use was independently associated with a reduced risk of SARS. | N. Rejects meta-analysis because heterogeneity; criticises Jefferson 2008 for doing one. Comments that findings in SARS may not transfer to influenza setting. |
| 1. Boulos et al. (2023) | Efficacy of face masks for reducing transmission of SARS-CoV-2 (HCW and community) | N | N | Rapid systematic review by Fellows of Royal Society (interdisciplinary). Includes non RCT evidence, talks of mechanisms. Few RCTs hence observational studies included. 35 in community (3 RCTs, 32 observational); 40 in HCWs (1 RCT, 39 observational). No meta-analysis. No ROB tool used but narrative critique. | Most observational studies were at ‘critical’ risk of bias in at least one domain, often failing to separate the effects of masks from concurrent interventions. Raw count: 39/47 studies found that masks reduced infection and 16/18 found that mask mandates did. 7 observational studies found that respirators were more protective than surgical masks, while 5 found no statistically significant difference. | Y. Concluded that despite high ROB, masks work and mask mandates work, and respirators are better than masks. |
| 1. Calò et al. (2020) Italy | Protection of HCWs from SARS-CoV-2 | N | N | Scoping review with systematic search. No meta-analysis. Included 4 early descriptive studies of HCWs. No RCTs. No ROB tool used. | These early studies suggested that HCWs attributed their infection with SARS-CoV-2 to inadequate PPE (or not following the full procedure); two small descriptive studies suggested no benefit of respirators over masks. | N. Undertaken very early in the pandemic. Main focus is on describing the burden (i.e. level of risk to HCWs) |
| 1. Camargo et al. (2020) Brazil | Effectiveness of non-woven face mask to prevent COVID-19 infection in the general population | Y | N | Rapid systematic review, identified one RCT and two systematic reviews (Liang 2020 and Benkouiten 2014). No meta-analysis. ROB2 used for the RCT. | Results presented in disaggregated form. The RCT and one systematic review suggested a benefit, the other systematic review suggested no benefit. | N. Early and brief review. |
| 1. Chaabna et al. (2021) | Effectiveness of medical and cloth masks against resp infections in community settings | Y | Y | 12 studies: 10 RCTs, 1 cohort, 1 case-control. All studies combined in a single meta-analysis, plus additional meta-analyses by outcome measure (CRI, ILI, serology). | Masks protective: OR 0.66 (0.54–0.81). | Oddly, they used GRADE only to classify all RCTs as high quality and all observational studies as low quality. No ROB assessment beyond this was undertaken. |
| 1. Chen et al. (2022) China | Masks against respiratory viral infections (community and HCW) | Y | Y | 31 studies: 6 RCTs, 7 observational cohort, 18 case-control. Three separate meta-analyses by study design. Cochrane ROB for RCTs, NOS for observational (no narrative justification). | Across 6 RCTs: masks reduced incidence of new cases with odds ratio, OR, 0.66 (0.5-0.88). Across 7 cohort studies: masks reduced incidence of new cases OR 0.31 (0.22-0.44). Across 18 case-control studies: masks reduced incidence of new cases OR 0.36 (0.26-0.48). | Y. Non-RCT designs tended to produce larger effect sizes. Used Newcastle-Ottawa ROB tool but didn’t seem to exclude the poorer quality studies from meta-analysis. |
| 1. Chen et al. (2025) China | Main question is link between *adherence to masking* and respiratory infections | N [?] | Y | Massive study – 70 countries, 448 studies, 654 datasets (105 on acceptability of mask wearing, 491 on mask wearing in public places, 84 on correct mask-wearing), plus 88 pages of supplementary tables. All 654 datasets included in a meta-analysis. Claimed to use AHRQ quality scale but reference links to an editorial on a different topic. Results column lotalic like they used Newcastle-Ottawa (NOS) and gave a total score out of 9 (no narrative justification). | In generalised linear models (GLM), a higher rate of mask-­wearing in public settings was significantly associated with lower COVID-­ 19 incidence (*β*= −265.2634, p=0.0039), COVID-­ 19 deaths (*β*=−2.037, p=0.0002) and other COVID-­ 19 pandemic-related deaths (*β*=−0.8324, p=0.0004). Greater adherence to masking was associated with larger effect sizes. | Y. Novel and complex study, but the underlying primary studies are mostly of VERY low quality! Mostly it’s about adherence. I am not persuaded that the summary statistics on incidence/death are valid (and certainly not to 4 decimal places!). |
| 1. Chou and Dana (2023), USA | Efficacy of masks for prevention of SARS-CoV-2 (HCW and community) | N | N | Update of living review that started out as a rapid review but changed to using full SR methods. In total, 24 studies on SARS-CoV-2, 11 in community, 13 in HCWs. 3 RCTs, 21 observational. In this update, they excluded any study based on self-reports (which had been included in previous versions). Study quality assessed using tool adapted from US Preventive Task Force. | Evidence for mask efficacy in community settings was judged overall “low to moderate”. N95s in community settings: “insufficient evidence”. In HCWs, N95 v surgical, any mask v no mask, and consistent v inconsistent mask use, all judged “insufficient evidence”. | Equivocal. Commented on the poor strength of evidence from many of the studies, and questioned the findings of the Loeb 2022 RCT in HCWs on the grounds of heterogeneity between sites and the possible explanation that workers could have caught the virus in the community. |
| 1. Chu et al. (2020) Canada | Physical interventions against respiratory coronaviruses (SARS, MERS, SARS-CoV-2). HCW and community. | N | Y | 39 mask studies (29 unadjusted, 10 adjusted); meta-analysis was limited to 30 comparative studies (25 cohort, 5 case-control: these were combined). No RCTs found. Most studies used lab-confirmed symptomatic infections. NOS (no narrative justification). | In community settings, masks reduced the risk of infection (RR 0.56, 95% CI 0.40–0.79). In healthcare settings, masks and respirators reduced the risk of infection by even more (RR 0.30, 95% CI 0.22–0.41), perhaps because greater use of N95 respirators. Respirators were, overall, 96% effective (adjusted OR 0.04, 95% CI 0.004–0.30) compared with masks, which were 67% effective (aOR 0.33, 95% CI 0.17–0.61). Unadjusted studies gave similar results to adjusted studies. | Y, Y. Newcastle-Ottawa 9-point ROB scale used, but didn’t exclude the poorer quality studies from meta-analysis. Many primary studies were small follow-up studies of HCWs in single hospitals after exposure to SARS or MERS. Authors speculate that community masking may be less efficacious due to self-inoculation from touching mask with contaminated hands (Sup p19) |
| 1. Coclite et al. (2021) Italy | Effectiveness of face masks in the community to prevent “COVID-19 or similar pandemic” | Y | Y | 35 studies: 3 RCTs, 10 comparative observational, 13 predictive models, 9 lab studies. Meta-analyses split by study design and (separately) setting. Cochrane ROB for RCTs, NOS for observational (no narrative justification). | Estimates of cluster-RCTs were in favour of masks but not at statistically significant levels (adjusted OR 0.90, 95% CI 0.78–1.05). Modelling suggests very large effect if population coverage is near-universal. Mask filtration efficacy depends on materials. | N. Used Cochrane ROB for RCTs, Newcastle-Ottawa for observational studies and QUADRIAC for modelling studies. |
| 1. Collins et al. (2021) USA | Respirators v medical masks for infection prevention in HCWs | Y | Y | 4 RCTs, 4 observational (3 case-control, 1 retrospective cohort). Meta-analysis combines study types. Cochrane ROB for RCTS, no tool used for observational (no narrative justification). | N95s significantly better than medical masks against ILI, non-influenza viral infection, SARS and COVID-19, and lab-confirmed respiratory viral infection, as a result of including case-control studies. | Y. Took no account of targeted use of respirators. |
| 1. Cowling et al. (2010) Hong Kong | Public health interventions for influenza prevention (HCW and community) | N | N | 6 HCW studies: 2 RCTs (respirators v masks, masks v none), 3 cross-sectional (masks part of wider protection package), 1 observational (from 1918, included PPE and “open wards” i.e. patients nursed outside). 5 community studies: 4 RCTs, 1 observational. 1 experimental volunteer study. No ROB tool used. | No meta-analysis done because of heterogeneity. RCTs were largely negative (no significant effect of masks in the community, no significant benefit of respirators over masks). | N. Meta-analysis deemed inappropriate due to heterogeneity of studies. |
| 1. Crespo et al (2023) | Efficacy of face masks in preventing COVID-19 transmission in real-world settings | N | N | Authors claim 48 studies, 21 in healthcare settings (but numbers don’t add up). 1 RCT, 5 case reports, 1 quasi-experimental, 6 cohort, 9 case-control, 22 cross-sectional. Joanna Briggs checklists but no results. | 44/48 studies showed that mask wearing reduced the risk of transmission of COVID-19. Authors concluded that the evidence “overwhelmingly supports that using face masks is effective at slowing or preventing the transmission of SARS-CoV-2 infection in a variety of real-world settings”. | Y. Preprint. Highly unusual selection of studies, many not included in other reviews, some anecdotal reports. Was never published in peer-reviewed journal. |
| 1. Dugré et al. (2020) Canada | Umbrella review of masks in community and HCWs | Y | N | 11 previous SRs were used to identify 18 RCTs (12 in community, 6 in HCWs). Meta-analysis of those studies. Cochrane ROB (online Appendix with these assessments is not available). | In community studies, no effect overall but “in the 2 trials that most closely aligned with mask use in real-life community settings”, there was a significant risk reduction in influenza-like illness (RR 0.83 [0.69-0.99]). No overall effect in household studies. In HCWs, surgical masks were superior to cloth masks for preventing ILI (RR 0.12 [0.02-0.98]), and N95 masks were likely superior to surgical masks for preventing ILI (RR 0.78 [0.61-1.00]) and CRI (RR 0.95 [0.90-1.00]). | N (masks), Y (N95s). Combined the targeted use of N95s with non-targeted use. |
| 1. Floriano et al. (2024) | Masking to prevent COVID-19 infection in HCWs and community (observational only) | N | Y | Observational studies only. 2 cohort studies and 7 case-control studies (all combined). ROBINS-I plus GRADE, but no narrative to justify decisions. | In cohort studies, wearing a cloth mask decreased risk of COVID-19 infection by 21% (RD −0.21 [−0.34 to −0.07]. In case-control studies, wearing a surgical mask reduced the chance of COVID-19 infection (OR 0.51 [0.37-0.70]), as did wearing an N95 respirator mask (OR 0.31 [0.20-0.49]). | Y. Quality of evidence was assessed as low in all cases.. |
| 1. Ford et al. (2021) Switzerland | Mask mandates for controlling respiratory disease outbreaks (community) | N | N | 21 observational (ecological) studies of mask mandates. Some were pre-post design, some followed the moving average or slope of cases (with lag). Comparisons with health system average or sometimes with adjacent settings without mandates. Used a bespoke adaptation of NOS to address ecological studies. | Data presented in disaggregated form in a table. All 21 studies showed some positive effect of mask mandates on (e.g.) R_t_ or some other measure of “flattening the curve”. Concluded that community mask policies may reduce population-level burden of SARS-CoV-2. | Y. Strong criticism of many ecological studies of masks from this team (whose own research focus is RCTs of handwashing). |
| 1. Gholami et al. (2021) United Arab Emirates | COVID-19 infection in HCWs and its correlates | N | N | 28 studies, mostly surveys of HCWs. No RCTs. No ROB tool used. | “Improper PPE” was correlated with increased infection risk in HCWs (also correlated was low levels of handwashing and various demographic risk factors and comorbidities). | Equivocal. Very little mention of masks, no meta-analysis. |
| 1. Greenhalgh et al. (2024) UK | Masks and respirators in prevention of respiratory infections (HCWs and community) | Y | N | 20 RCTs (14 community, 6 HCWs), plus non-exhaustive overview of observational evidence. New meta-analysis of RCTs but no new meta-analysis of observational evidence. No ROB tool used. | Community RCTs: significant effect of masks but only for ILI. HCWs: significant effect of masks v no masks and of respirators v masks, if targeted use of respirators analysed separately from continuous use. Observational studies summarised narratively: significant effect of masks, which is notable because study flaws would tend to bias towards the null. | Y, Y. Raised questions about inconsistencies in one RCT (Loeb 2022). |
| 1. Hajmohammadi et al. (2023) | Masks and other interventions to reduce spread of COVID-19 (HCW and community) | N | Y | Meta-analysis of case-control studies. 14 studies – 8 in HCWs and 6 in community. NOS (no narrative justification). | PPE or any type of mask was associated with reduction in risk of COVID-19 (OR 0.44 [0.29, 0.65]; *I*2 = 85.21%). In the HCW subgroup, the protective effect had a combined OR of 0.33 (0.15,0.73). Six studies found protective effects of masks in non‑HCWs (OR 0.58 [0.31, 1.06]). | Y (N95s and any mask for HCWs), N (community). Newcastle-Ottawa ROB |
| 1. Iannone et al. (2020) | Respirators and masks for protection of HCWs during COVID-19 | Y | N | 4 RCTs identified (none in COVID-19). Meta-analysis. Cochrane ROB (no narrative justification). | N95 respirators can prevent 73 (46–91) more clinical respiratory infections per 1000 HCWs compared to surgical masks. A protective effect of N95 respirators in laboratory-confirmed bacterial colonization (RR = 0.41 [0.28–0.61]) was also found. | Y (N95s). Authors judged quality of evidence low. |
| 1. J. Li et al. (2021) China | Respirators and masks against respiratory infection in healthcare workers | Y | Y | Network meta-analysis of 5 RCTs and 26 observational studies. Cochrane ROB for RCTs, NOS for observational (no narrative justification). | Network meta-analysis showed overall OR 0.38 (0.21-0.69) for becoming infected if wearing face mask, but ordinary pooled meta-analysis did not. The single community study showed less of an effect than the healthcare studies. No overall difference between medical masks and respirators. | Y. No risk of bias assessment except funnel plot for publication bias. Combined both MacIntyre 2013 arms (targeted and continuous) |
| 1. Jefferson et al. (2008) UK | Physical interventions against respiratory infections (HCW and community) | N | Y | Includes 7 cohort studies, all in healthcare workers (HCWs), in which masking was a component, plus a meta-analysis of 6 case-control studies in HCWs, all in SARS, which isolated out the effect of masking. No RCTs found. Newcastle-Ottawa (NOS); no narrative justification. | Meta-analysis showed that wearing masks appeared to confer significant protection against respiratory infection compared to no masks (OR 0.32 [0.25 to 0.40]) and N95 masks gave even greater protection (OR 0.09 [0.03-0.30]). NB There WAS one RCT (Murphy 1981) but authors misclassified it as a case-control. | Y, Y. Newcastle-Ottawa risk of bias (ROB) 9-point scale used for non-RCT studies; early version of Cochrane ROB tool used for RCTs. ROB judged low if 0 or 1 item was considered “inadequate”; medium if 2-3 were, and high if more than 3 were. |
| 1. Jefferson et al. (2020) UK (Update) | Physical interventions against respiratory infections (HCW and community) | Y | N | 14 RCTs. Medical masks (7 in community, 2 in HCWs); respirators v masks (1 in community, 4 in HCWs). Meta-analysis. No observational studies included (unlike in Jefferson 2008 – see below). Cochrane ROB (some narrative justification given in text). | No statistically significant effect of masks over no masks (e.g. for lab-confirmed influenza, RR 0.91 [0.66-1.26]), or of respirators over masks (e.g. for lab-confirmed influenza RR 1.10 [0.90-1.34]. | N. All RCTs of medical masks were combined whether in community or HCWs; all RCTs of respirators v masks combined. The targeted and non-targeted arms of MacIntyre 2013 were combined (see p 144). |
| 1. Jefferson et al. (2023) | (Update) Physical interventions against respiratory infections (HCW and community) | Y | N | 17 RCTs total. 12 of masks v no masks (2 HCW, 10 community), 5 of respirator v mask (4 HCW, 1 community). No observational studies included. Meta-analysis. Cochrane ROB (some narrative justification given in text). | No statistically significant effect of masks over no masks (RR 1.01 [0.72-1.42] for lab-confirmed influenza or COVID-19, or of respirators over medical masks (RR 1.10 [0.90 to 1.34]). | N, N. Same problem as with Jefferson 2020. Same findings. Community and HCW not separately analysed; targeted respirator use not separately analysed. See page 175: the two arms of MacIntyre 2013 (targeted and continuous) are combined in the same meta-analysis. |
| 1. Juneau et al. (2022) | Narrative review from public health perspective looking for “lessons from past pandemics”. | N | N | Wide-ranging review with major focus on cost-effectiveness. Considered school closures, lockdowns, contact tracing, handwashing plus masks. Included systematic reviews of RCTs (Smith 2015, MacIntyre and Chugtai 2015) but no new analysis of RCTs. No ROB tool used. | “The effectiveness of hand-washing and face masks was supported by randomized trials. These measures were highly cost-effective.” No evidence for the other measures. No meta-analyses. | Y. Limited detail given on how conclusions were reached. Unusually, did not use Cochrane-style ROB tools or a PRISMA statement. |
| 1. Kim et al. (2022) South Korea | To evaluate the  comparative effectiveness of N95, medical and non‐medical masks against respiratory virus infection (HCW and community) | Y | Y | 35 RCTs and observational studies (12 RCTs, 23 observational = 8 case-control, 6 cohort, 1 cross-sectional, 1 non-randomised comparative, plus case reports). 27 HCW, 8 community. Cochrane ROB (v2) for RCTs, ROBINS-I for observational. Detailed justification of judgements given in separate tables. | High compliance to mask‐wearing conferred a significantly better protection (odds ratio [OR], 0.43; 95% confidence interval [CI], 0.23–0.82) than low compliance. N95 or equivalent masks were the most effective against coronavirus infections (OR, 0.30; CI, 0.20–0.44) consistently across subgroup analyses of causative viruses and clinical settings | Y. Used ROB2 for RCTs, ROBINS-I for observational. Authors comment that only one RCT was available for COVID-19. Supplementary data is 85 pages long. |
| 1. Kunstler et al. (2022) Australia | Masks v respirators in HCWs – prevention of SARS-CoV-2 infection and adverse effects | Y | y | 1 RCT, 20 observational studies. For efficacy, 1 RCT and 11 observational. For adverse effects, 11 (10 additional) observational studies (10 cohort, 1 case control), of which 8 contributed to a meta-analysis. Cochrane ROB (v2) for RCTs, ROBINS-I for observational (no narrative justification). | Most studies had high risk of bias. No sig diff between respirator and surgical mask (OR 0.85, [0.72-1.01]). HCWs experienced significantly more headaches (OR 2.62 [1.18-5.81]), respiratory distress (OR 4.21, [1.46-12.13]), facial irritation (OR 1.80 [1.03-3.14]) and pressure-related injuries (OR 4.39 [2.37-8.15]) when wearing respirators compared to surgical masks. | N. Concluded that added benefits of respirators are unproven and adverse effects commoner. BUT did not split targeted from non targeted use. |
| 1. Li et al. (2022) China | 2 questions: a) efficacy of masks, b) factors affecting adherence (HCW and community) | Y | Y | 8 RCTs (1 HCW, 7 community) plus 78 other studies (mostly surveys of perceptions, intentions and practices). Cochrane Handbook for RCTs, Joanna Briggs for observational studies (no data, no narrative justification). | Meta-analysis of RCTs showed a significant protective effect of masks (OR 0.84 [0.71-0.99]). If duration more than 2 weeks, effect was more marked (OR 0.76 [0.66-0.88]). Meta-analysis of observational studies showed that, overall, 71% of respondents believed masks were effective, 68% “would” wear them, and 54% did wear them for preventing respiratory infections. | Y. Used Cochrane ROB for RCT bias assessment, Joanna Briggs for observational studies. |
| 1. Liang et al. (2020) Singapore | Respirators versus masks, any infection (HCW and community) | Y | Y | 6 RCTs (1 in HCWs, 5 in community), 15 observational studies (2 cohort, 13 case-control). Meta-analyses of all 21 studies, split by HCW/community, Asian/non-Asian and virus type (influenza / SARS / COVID-19). In most analyses, study designs were pooled (RCTs analysed along with non-RCTs), but one separate analysis was done. Jadad ROB tool for RCTs, NOS for observational (no narrative justification). | In the combined analysis, masks were significantly protective (OR 0.35 [0.24-0.51]). OR for HCWs was 0.20 (0.11-0.37) and non-HCWs 0.53 (0.36-0.79). Masking in Asia (OR 0.31) appeared higher than in Western countries (OR 0.45). Masks had a protective effect against influenza viruses (OR = 0.55), SARS (OR = 0.26), and SARS-CoV-2 (OR = 0.04). Masks appeared to have a greater effect in cluster RCTs (OR 0.65 [0.47–0.91]) and observational studies (OR 0.24 [0.15–0.38]). | Y. Used Cochrane ROB and Newcastle-Ottawa scale for RCTs and observational studies respectively. RCT evidence independently showed a significant effect in both community and HCWs; Asian studies showed greater effect than Western ones |
| 1. Long et al. (2020) China | Respirators versus masks for prevention of influenza in HCWs | Y | N | 6 RCTs (all in HCWs), meta-analysis. No other studies. Cochrane ROB (no narrative justification). | No significant benefit of N95 respirators against lab-confirmed viral infection or influenza or respiratory infection (e.g. in lab-confirmed respiratory infection (RR= 0.74 [0.42-1.29]), but significant advantage over medical masks on bacterial colonization (RR= 0.58 [0.43-0.78]) | N in 2/3 analyses. Did not separate targeted use of respirators from continuous use. |
| 1. Lu et al. (2023) Canada | Masks and respirators in protection of HCWs (umbrella meta-analysis) | N | N | Summarised 6 previous meta-analyses of RCTs in HCWs. AMSTAR-2 for meta-analyses (no narrative justification). | Produced a summary statistic that implied significant advantage of respirators, based on doing a forest plot of 6 previous meta-analyses and then summing the means. | Y. Findings not trustworthy (forest plot of meta-analyses appears meaningless). Paper contains errors (e.g. Kunster has no RCTs against a respiratory infection outcome). |
| 1. MacIntyre and Chughtai (2020) | Masks and respirators for prevention of respiratory infections (HCW and community) | N | N | Rapid systematic review. 19 RCTs (8 in community, 6 in HCWs and 5 of source control where sick person wears mask to protect others). No meta-analysis (findings presented as disaggregated primary studies). No formal ROB tool used but narrative critique provided in text. | In the community, masks appeared to be effective with and without hand hygiene, and both together were more protective. In HCWs, respirators, if worn continually during a shift, were effective but not if worn intermittently. Medical masks were not effective, and cloth masks even less effective, in HCWs. When masks were used by sick patients, RCTs suggested protection of well contacts. | Y, Y. Disaggregated intermittent from continuous respirator use. |
| 1. Mendez-Brito et al. (2021) Germany | To evaluate which NPIs have been more or less effective at controlling the COIVD-19 pandemic (real-world studies) | N | Y | 34 ecological studies covering masks as well as school closures, lockdown etc. 7 studies of mask mandates. Bespoke tool for ecological studies (adapted from Dufault & Klar 2011). No narrative justification. | 6 of 7 studies of mask mandates showed a significant effect. In 1st wave of COVID-19, school closing was the most effective NPI, followed by workplace closing, business and venue closing and public event bans. Public information campaigns and mask wearing requirements were also effective. Early implementation improved efficacy; stringency did not. | Y. Good example of a systematic review of mandates as opposed to masks. |
| 1. Nanda et al. (2021) UK | Masks in prevention of respiratory infection (HCW and community) | Y | Y | 12 RCTs (11 community, 1 HCWs, 11 included in a meta-analysis), 1 preclinical (hamster) study, 1 observational cohort (Y Wang in Beijing). Cochrane ROB for RCTs, ROBINS-I for observational, OHAT for preclinical (no narrative justification). | Surrogate and observational study evidence showed significant benefit of surgical mask v no mask, but meta-analysis of RCT evidence did not. | N (RCT), Y (observational). All RCTs combined wrt outcome measure, not subdivided by setting |
| 1. Offeddu et al. (2017) Singapore | Efficacy of masks and respirators for HCW protection against respiratory infection | Y | Y | 5 RCTs; 20 observational studies. In SARS and influenza. Split by outcome (ILI, CRI, lab-confirmed) but cohort and case-control combined. Cochrane ROB for RCTs, NOS for observational (no narrative justification). | Meta-analysis suggested protective effect of masks and respirators against clinical respiratory illness (CRI) (risk ratio [RR] = 0.59 [0.46–0.77]) and influenza-like illness (ILI) (0.34 [0.14–0.82]). Compared to masks, N95 respirators conferred superior protection against CRI (RR = 0.47 [0.36–0.62]) and laboratory-confirmed bacterial (RR = 0.46 [0.34–0.62]), but not viral infections or ILI. Meta-analysis of observational studies suggested protective effect of masks (OR 0.13 [0.03–0.62]) and respirators (OR = 0.12 [0.06–0.26]) against SARS. | Y, Y. Continuous use of respirators not separated from targeted use |
| 1. Ollila et al. (2022) Finland | Masks in reducing spread of respiratory infections (HCW and community) | Y | N | 18 RCTs (8 community, 10 HCW). No non-RCT studies. Meta-analysis. Cochrane ROB (no narrative justification). | Study heterogeneity was high. Follow-up ranged from 4 days to 19 months. No statistically significant association over all studies using unadjusted effect estimates (RR 0.977 [0.858–1.113), subgroup analyses revealed a significant effect in the adult subgroup (RR = 0.88 [0.79–0.98]) and in community settings (RR = 0.89 [0.81–0.98]). | N (but Y in some subgroups). Unusually, household studies were analysed with HCWs as “non-community” on the grounds that these are high-risk settings. Studies of children often documented co-sleeping (unmasked) with parents. |
| 1. Peccin et al. (2022) | Efficacy, safety, use and re-use of medical masks in preventing infection with COVID-19 (HCW and community) | N | N | 10 studies, of which 5 involved masks or respirators: 3 observational (cross-sectional), 1 laboratory, 1 simulation in a clinical setting. No RCTs. Joanna Briggs checklists but no results. | N95 respirator along with face shield appeared to be the most effective protection for HCWs. | Y. Unusual selection of studies, mostly laboratory and with several including 3D-printed face shields and eye protection, so effect of masks hard to disaggregate. |
| 1. Perski et al. (2022) | Interventions to improve personal protective behaviours (includes brief review of face mask efficacy) | N | N | 12 RCTs, all community. Cochrane ROB (domain scores given but not individual items, no narrative justification). | Disaggregated findings given: “three studies reporting positive effects, two studies reporting no difference, one study reporting negative effects, and six studies with indeterminate results” | Assessment of mask efficacy was a tiny part of this wider study which was mainly about interventions to increase adherence. |
| 1. Peters and Farhadloo (2023) Canada | Impact of NPIs (mandates) on transmission of COVID-19 | N | Y | 44 observational (all ecological) studies of NPI mandates, including 7 studies of mask mandates. NOS (no narrative justification for these judgements but some brief narrative commentary in supplementary materials). | Masks were associated with mitigative effects on both cases (–2.76 per 100,000) and deaths (–0.19 per 100,000). Showed the more stringent and the longer the mandate is in place the bigger the effect. | Y. “When assessing the impact of NPIs, considering the duration of effectiveness after implementation has paramount significance.” |
| 1. Ramaraj et al. (2020) UK | Masks v respirators for protection of HCWs | N | N | 9 studies: 3 surrogate exposure; 6 clinical studies (4 RCTs, 2 observational). No meta-analysis. CASP checklists. | Surrogate exposure studies demonstrated the superiority of respirators. The RCTs showed that continuous respirator use was more effective than intermittent use, which was no more effective than ordinary masks. | Y (N95s, continuous). GRADE criteria used to assess clinical studies |
| 1. Rohde et al. (2020) Ireland | Masks worn in community settings for reducing transmission of SARS-CoV-2 | N | N | 7 observational studies (6 community, 1 households), all in COVID-19. 2 case-control, 3 cross-sectional, 2 retrospective cohort. No RCTs. Forest plot but no meta-analysis summary statistic. Joanna Briggs checklists but no results. | Face masks reduce the risk of SARS-CoV-2  infection; however, all studies were at high risk of bias and the quality of the evidence was low | Y. Undertaken too early to capture many studies in COVID-19 |
| 1. Samaranayake et al. (2020) | Efficacy of masks and respirators for HCW protection in dental settings | N | N | Qualitative synthesis (16 mask studies): 6 studies comparing surgical masks and respirators (3 RCTs, 3 simulation / experimental models). 7 studies of fit factor: 1 RCT and 6 simulation experiments of face-seal factor comparing fit-tested respirators and surgical masks. 3 studies of face mask efficacy with wear-time. No meta-analysis. Cochrane ROB for RCTs, NOS for lab studies (this was not what it was designed for). | Wearing layered, face-fitting masks or respirators and protective-eyewear can limit the spread of infection among HCWs. Specifically, combined interventions such as a face mask and a face shield, better resist bioaerosol inhalation in dental contexts than either alone. The prolonged and over-extended use of surgical masks compromise their effectiveness. | Y. Included studies of jets and aerosolization from drilling teeth (this changes the dynamics, making face shields and goggles more relevant, for example). |
| 1. Sami et al. (2023) Iran | Face masks for viral illness prevention in healthcare settings | N | Y | Systematic review and meta-analysis of 6 studies (5 observational studies, plus one laboratory RCT in which participants were randomised to mask or no mask for an exhaled breath study). Jadad for RCTs, NOS for observational (no narrative justification). All combined in one meta-analysis. | Wearing a face mask significantly reduced the risk of contracting a respiratory viral illness in hospital settings, with pooled OR 0.11 (0.04 to 0.33). | Y. Jaded Scale for RCTs, Newcastle-Ottawa for observational ROB. |
| 1. Schoberer et al. (2022) Austria | Masks and other NPIs for protection of HCWs against SARS-CoV-2 | N | Y | Rapid Cochrane review.  16 systematic reviews, from which they extracted primary studies and then looked for additional ones. Final dataset for mask component was 7 observational studies (1 of which identified no cases). No RCTs. AMSTAR-2 for reviews, NOS for observational studies (no narrative justification). Diff study designs combined in meta-analysis. | Masking (with N95, surgical or unspecified) significantly reduced risk of HCW infection with SARS-CoV-2 when all groups combined (OR 0.16 [0.05-0.55]). Estimate for N95s, from 3 studies, was 0.08 (0.01-0.65). | Y. Used Newcastle-Ottawa ROB tool. |
| 1. SeyedAlinaghi et al. (2023) | Face masks in preventing transmission of COVID-19 | N | N | Review of reviews. Identified 28 previous reviews. No ROB tool used. | Most reviews found that face masks are beneficial against viral respiratory infections, such as COVID-19. Mask efficacy depends on the material, layers, fitting on the face and user compliance. N95 respirators had maximum efficacy, especially when used continuously. | Y. But derivative (i.e. summarised previous reviews). |
| 1. Sharma et al. (2020) | Cloth masks for prevention of respiratory infections (community) | N | N | 2 RCTs, 1 non-randomised comparative study, 9 laboratory studies of filtration efficacy. No meta-analysis. Cochrane ROB (v2) for RCTs, STROBE for observational. | Cloth face masks showed less efficacy in source control than medical masks. Filtration efficacy varied with type of material, number of layers, humidity and fit. | Equivocal. Early review, few included studies. |
| 1. Smith et al. (2015) | NPIs in preventing transmission of influenza in adilts | N | N | For mask component, 5 RCTs. CASP and SIGN checklists (no narrative justification). | On the basis of the few available studies at the time, concluded that face masks have limited effectiveness alone against spread of influenza in the community but may be effective when combined with hand hygiene. They may reduce secondary transmission in households. | N. Observational studies are described very briefly in text; review is mainly focused on RCTs. |
| 1. Smith et al. (2016) Canada | Respirators v masks for prevention of infections in HCWs | Y | Y | 3 RCTs, 1 cohort, 2 case-control, 23 surrogate exposure studies. Meta-analyses split by study design. Cochrane ROB for RCTs, NOS for observational (no narrative justification). | No significant difference between N95 respirators and surgical masks in laboratory-confirmed respiratory infection (RCTs: OR 0.89 [0.64–1.24]; cohort study: OR 0.43 [0.03–6.41]; case-control studies: OR 0.91 [0.25–3.36]). But in surrogate exposure studies, N95 respirators were associated with less filter penetration, less face-seal leakage and less total inward leakage under laboratory experimental conditions, compared with surgical masks | N. Demonstrated a major discrepancy between laboratory evidence (which supported superiority of respirators over masks) and clinical studies (which did not appear to) |
| 1. Soleman et al. (2023) Japan | Masks and respirators to protect HCWs against COVID-19 | N | N | 47 papers comprising: 7 reviews (which covered various RCTs), 7 cohort, 9 case-control, 15 cross-sectional studies, 4 before and after, 4 case series, and 1 modelling study. No new meta-analysis. ACROBAT-NRSI (an early ROB tool for obs studies). | Comparisons very generic and, in relation to masks, mostly summarised the findings of other reviews (in particular, saying that respirators offer similar protection to medical masks). But overall, this reads as a very rapid review lacking depth. | Equivocal. Very limited detail given on how papers were selected or assessed. Observational studies assessed using ACROBAT-NRSI risk of bias tool. |
| 1. Tabatabaeizadeh (2021) | Masks in preventing airborne transmission of SARS-CoV-2 | N | N | 4 observational studies (retrospective case control studies of staff exposed to infected patients). No RCTs. NOS use claimed (but no results given). | All studies showed that unprotected HCWs were more likely to become infected. 2 studies showed respirators provided greater protection. | Y. Early rapid review. |
| 1. Talic et al. (2021) Australia | Impact of public health measures on incidence of COVID-19, transmission of SARS-C0V-2 and mortality from COVID-19 | Y | Y | Multiple public health measures. Face mask component identified 1 RCT, 6 comparative observational and 5 non-comparative observational studies. The one RCT was included in the meta-analysis with cohort and case-control studies. Cochrane ROB (v2) for RCTs, ROBINS-I for observational (no justification of judgements on items but ‘limitations’ column in table of included studies). | In the comparative studies, overall pooled analysis showed a 53% reduction in covid-19 incidence (0.47 [0.29-0.75]), but heterogeneity between studies was substantial and risk of bias varied. Non-comparative studies also report large effects of masks. E.g. A natural experiment across 200 countries showed 45.7% less covid-19 related mortality in countries where mask wearing was mandatory (Leffler). | Y. Little detail given on how each study was assessed (except for saying ROBINS-I tool used), but ALL 11 studies were assigned at least moderate risk of bias and 2 were ‘serious or critical’. |
| 1. Tran et al. (2021) Japan | Masks in prevention of respiratory infectious diseases (HCW and community) | Y | N | RCTs only. Network meta-analysis: 16 RCTs total. 11 community, 5 HCWs. Cochrane ROB (no narrative justification). | Fit-tested respirators were significantly more protective than no masks (RR 0.67 [0.38–1.19]), as were non-fit tested respirators (slightly higher RR). Using *pair-wise* meta-analysis, a significant effect of face masks was shown (RR 0.83 [0.71–0.96]), but effect was not significant if used standard (pooled) meta-analysis. | Y, Y. Main distinguishing feature is the network approach. All RCTs were combined with respect to outcome measure, not subdivided by setting. |
| 1. Wang et al. (2020) Singapore | Surgical masks for prevention of respiratory infections (community) | Y | Y | 5 RCTs (in separate meta-analysis) plus 10 observational studies also in a meta-analysis (7 cross-sectional, 1 retrospective case-control, 1 nested case-control, 1 prospective cohort). Split by setting and by outcome. Cochrane ROB for RCTs, NHLBI for observational (no narrative justification). | A modest but non-significant protective effect of masks on ARI incidence was observed (pooled OR 0.96 [0.8–1.15]). Subgroup analysis according to age group, outcome ascertainment and different non-healthcare settings showed no significant effect. | N. ROB assessed using Cochrane tools and STROBE for observational studies. |
| 1. Xiao et al. (2020) Hong Kong | Masking and other measures in influenza (community settings) | Y | N | 10 RCTs of masks in community. Also looked at hand washing and surface cleansing. Meta-analysis. No ROB tool used. | Pooled meta-analysis showed no significant effect of masks over no masks (e.g. for face mask alone, RR = 0.78 [0.51-1.20]). | N. Focused only on influenza |
| 1. Y. Li et al. (2021) Hong Kong | Masks against COVID-19 infection. HCW and community. | N | Y | 6 case-control studies (5 in HCWs, 1 in community). NOS (no narrative justification). | Masking was associated with a significantly reduced risk of COVID-19 infection (OR 0.38 [0.21-0.69]. In HCWs, masks reduced risk of infection by nearly 70%. Sensitivity analysis showed that the results were robust. | Y, Y. Included extensive search of Chinese databases. Studies were weighted for quality. |
| 1. Yanfei Li et al. (2021) | Masks in preventing spread of respiratory viruses, healthcare + community | N | N | 21 RCTs, 9 systematic reviews. Cochrane ROB for RCTs, AMSTAR-2 for systematic reviews. No meta-analysis, used evidence mapping instead (bubble plots of individual studies). | Results given in disaggregated form: “Overall, masks versus usual practice, 10 of 14 RCTs and 5 of 6 SRs were classified as “beneficial” or “probably beneficial”. Furthermore, regarding N95 respirators versus medical masks, 3 of 6 RCTs were classified as “beneficial”.” Also commented that systematic reviews showed little or no overall effect. | One of the RCTs was a duplicate so there were really only 20. Evidence mapping creates visuals using research populations, sample size /number of RCTs, the rating of conclusions, and quality assessment |
| 1. Yin et al. (2021) China | Masks and respirators for infection prevention in HCWs | Y | N | Network meta-analysis of 6 RCTs. HCWs only. Cochrane ROB (no narrative justification). | Respirator compared to medical mask: not significantly better (RR 0.67 [0.24-1.89]). Continuous wearing during the entire shift was significantly better than surgical masks (RR 0.46 [0.23-0.95]) for CRI. Networked analysis showed increasing efficacy moving from cloth🡪 surgical🡪targeted N95🡪continuous N95. | Y (N95, only if continuous). One of only two meta-analyses to split targeted versus continuous respirator use. |
| 1. Banholzer et al. (2022) Switzerland | What methods have been used in studies of NPIs in COVID-19 | N | N | This was a systematic review of methodologies, so no risk of bias assessment was done. Extensive search, identified 248 studies (RCTs, observational incl modelling). | Studies had used widely differing methodologies, used different terms in different ways. Statistical approaches varied (e.g. descriptive, parametric, counterfactual). | n/a. Highlights how difficult it is to summarise and make sense of these studies. |

Abboah-Offei, M., Salifu, Y., Adewale, B., Bayuo, J., Ofosu-Poku, R., & Opare-Lokko, E. B. A. (2021). A rapid review of the use of face mask in preventing the spread of COVID-19. *International journal of nursing studies advances*, *3*, 100013.

Aggarwal, N., Dwarakanathan, V., Gautam, N., & Ray, A. (2020). Facemasks for prevention of viral respiratory infections in community settings: A systematic review and meta-analysis. *Indian journal of public health*, *64*(6), 192-200.

Alhajaji, R., Al Sulaiman, K., Abdelwahab, O. A., Alfahmi, M., Abualenain, J. T., Asiri, S. I., Aljuhani, O., Alharbi, A., Alkofide, H., Ahmed, M., Saeedi, A. Y., Alturkistani, A., & Al-Jedai, A. (2024). Effectiveness of Face Mask for Prevention of Respiratory Tract Infection during Hajj: A Systematic Review and Meta-Analysis. *Am J Trop Med Hyg*, *111*(4), 914-923. <https://doi.org/10.4269/ajtmh.23-0667>

Alihsan, B., Mohammed, A., Bisen, Y., Lester, J., Nouryan, C., & Cervia, J. (2022). The efficacy of facemasks in the prevention of COVID-19: a systematic review. *MedRxiv*, 2022.2007. 2028.22278153.

Alkhalaf, A., Aljaroudi, E., Al-Hulami, M., Gaffar, B., & Almas, K. (2023). Efficacy of Surgical Masks Versus N95 Respirators for the Prevention of COVID-19 in Dental Settings: A Systematic Review. *Cureus*, *15*(4), e37631. <https://doi.org/10.7759/cureus.37631>

Banholzer, N., Lison, A., Özcelik, D., Stadler, T., Feuerriegel, S., & Vach, W. (2022). The methodologies to assess the effectiveness of non-pharmaceutical interventions during COVID-19: a systematic review. *European journal of epidemiology*, *37*(10), 1003-1024.

Barasheed, O., Alfelali, M., Mushta, S., Bokhary, H., Alshehri, J., Attar, A. A., Booy, R., & Rashid, H. (2016). Uptake and effectiveness of facemask against respiratory infections at mass gatherings: a systematic review. *International Journal of Infectious Diseases*, *47*, 105-111.

Bartoszko, J. J., Farooqi, M. A. M., Alhazzani, W., & Loeb, M. (2020). Medical masks vs N95 respirators for preventing COVID‐19 in healthcare workers: A systematic review and meta‐analysis of randomized trials. *Influenza and other respiratory viruses*, *14*(4), 365-373.

Barycka, K., Szarpak, L., Filipiak, K. J., Jaguszewski, M., Smereka, J., Ladny, J. R., & Turan, O. (2020). Comparative effectiveness of N95 respirators and surgical/face masks in preventing airborne infections in the era of SARS-CoV2 pandemic: A meta-analysis of randomized trials. *PloS one*, *15*(12), e0242901. <https://doi.org/10.1371/journal.pone.0242901>

Benkouiten, S., Brouqui, P., & Gautret, P. (2014). Non-pharmaceutical interventions for the prevention of respiratory tract infections during Hajj pilgrimage. *Travel Med Infect Dis*, *12*(5), 429-442. <https://doi.org/10.1016/j.tmaid.2014.06.005>

Bin-Reza, F., Lopez Chavarrias, V., Nicoll, A., & Chamberland, M. E. (2012). The use of masks and respirators to prevent transmission of influenza: a systematic review of the scientific evidence. *Influenza Other Respir Viruses*, *6*(4), 257-267. <https://doi.org/10.1111/j.1750-2659.2011.00307.x>

Boulos, L., Curran, J. A., Gallant, A., Wong, H., Johnson, C., Delahunty-Pike, A., Saxinger, L., Chu, D., Comeau, J., & Flynn, T. (2023). Effectiveness of face masks for reducing transmission of SARS-CoV-2: a rapid systematic review. *Philosophical Transactions of the Royal Society A*, *381*(2257), 20230133.

Calò, F., Russo, A., Camaioni, C., De Pascalis, S., & Coppola, N. (2020). Burden, risk assessment, surveillance and management of SARS-CoV-2 infection in health workers: a scoping review. *Infectious Diseases of Poverty*, *9*(05), 1-11.

Camargo, M. C., Martinez-Silveira, M. S., Lima, A. A., Bastos, B. P., Santos, D. L. D., Mota, S. E. C., Silva, R. B., & Toledo, I. P. (2020). Effectiveness of the use of non-woven face mask to prevent coronavirus infections in the general population: a rapid systematic review. *Cien Saude Colet*, *25*(9), 3365-3376. <https://doi.org/10.1590/1413-81232020259.13622020> (Eficácia da máscara facial (TNT) na população para a prevenção de infecções por coronavírus: revisão sistemática.)

Chaabna, K., Doraiswamy, S., Mamtani, R., & Cheema, S. (2021). Facemask use in community settings to prevent respiratory infection transmission: A rapid review and meta-analysis. *International Journal of Infectious Diseases*, *104*, 198-206.

Chen, C., Zhou, W., Qi, J., Chen, M., Yuan, Z., Miao, J., Yang, M., Chen, J., Shen, F., Cao, K., Qu, R., Jiang, D., Du, Y., Wu, X., You, Y., Yan, R., Zhu, C., & Yang, S. (2025). Adherence to mask-wearing and its impact on the incidence and deaths of viral respiratory infectious diseases: a systematic review, meta-analysis and modelling study. *BMJ Glob Health*, *10*(2). <https://doi.org/10.1136/bmjgh-2024-017087>

Chen, Y., Wang, Y., Quan, N., Yang, J., & Wu, Y. (2022). Associations Between Wearing Masks and Respiratory Viral Infections: A Meta-Analysis and Systematic Review. *Front Public Health*, *10*, 874693. <https://doi.org/10.3389/fpubh.2022.874693>

Chou, R., & Dana, T. (2023). Major Update: Masks for Prevention of SARS-CoV-2 in Health Care and Community Settings-Final Update of a Living, Rapid Review. *Ann Intern Med*, *176*(6), 827-835. <https://doi.org/10.7326/m23-0570>

Chu, D. K., Akl, E. A., Duda, S., Solo, K., Yaacoub, S., Schünemann, H. J., Chu, D. K., Akl, E. A., El-harakeh, A., Bognanni, A., Lotfi, T., Loeb, M., Hajizadeh, A., Bak, A., Izcovich, A., Cuello-Garcia, C. A., Chen, C., Harris, D. J., Borowiack, E.,…Schünemann, H. J. (2020). Physical distancing, face masks, and eye protection to prevent person-to-person transmission of SARS-CoV-2 and COVID-19: a systematic review and meta-analysis. *The Lancet*, *395*(10242), 1973-1987. <https://doi.org/10.1016/S0140-6736(20)31142-9>

Coclite, D., Napoletano, A., Gianola, S., Del Monaco, A., D'Angelo, D., Fauci, A., Iacorossi, L., Latina, R., Torre, G. L., & Mastroianni, C. M. (2021). Face mask use in the community for reducing the spread of COVID-19: a systematic review. *Frontiers in medicine*, *7*, 594269.

Collins, A. P., Service, B. C., Gupta, S., Mubarak, N., Zeini, I. M., Osbahr, D. C., & Romeo, A. A. (2021). N95 respirator and surgical mask effectiveness against respiratory viral illnesses in the healthcare setting: a systematic review and meta‐analysis. *JACEP Open*, *2*(5), e12582.

Cowling, B., Zhou, Y., Ip, D., Leung, G., & Aiello, A. (2010). Face masks to prevent transmission of influenza virus: a systematic review. *Epidemiology & infection*, *138*(4), 449-456.

Crespo, N., Fornasier, J., Dionicio, P., Godino, J., Ramers, C., & Elder, J. (2023). Effectiveness of Face Masks in Preventing COVID-19 Transmission in Real-World Settings: A Systematic Literature Review. *Research Square*. <https://doi.org/10.21203/rs.3.rs-2534269/v1>

Dugré, N., Ton, J., Perry, D., Garrison, S., Falk, J., McCormack, J., Moe, S., Korownyk, C. S., Lindblad, A. J., Kolber, M. R., Thomas, B., Train, A., & Allan, G. M. (2020). Masks for prevention of viral respiratory infections among health care workers and the public: PEER umbrella systematic review. *Can Fam Physician*, *66*(7), 509-517.

Floriano, I., Silvinato, A., Bacha, H. A., Barbosa, A. N., Tanni, S., & Bernardo, W. M. (2024). Effectiveness of wearing masks during the COVID-19 outbreak in cohort and case-control studies: a systematic review and meta-analysis. *Jornal Brasileiro de Pneumologia*, *49*, e20230003.

Ford, N., Holmer, H. K., Chou, R., Villeneuve, P. J., Baller, A., Van Kerkhove, M., & Allegranzi, B. (2021). Mask use in community settings in the context of COVID-19: A systematic review of ecological data. *eClinicalMedicine*, *38*. <https://doi.org/10.1016/j.eclinm.2021.101024>

Gholami, M., Fawad, I., Shadan, S., Rowaiee, R., Ghanem, H., Khamis, A. H., & Ho, S. B. (2021). COVID-19 and healthcare workers: A systematic review and meta-analysis. *International Journal of Infectious Diseases*, *104*, 335-346.

Greenhalgh, T., Helm, R. K., Poliseli, L., Ratnayake, S., Trofimov, A., & Willliamson, J. (2025). Protocol: A Systematic Review+ (SR+) to combine associative and mechanistic evidence on the efficacy of face masks in reducing transmission of respiratory diseases. *BMC Systematic Reviews (in press)*.

Greenhalgh, T., MacIntyre, C. R., Baker, M. G., Bhattacharjee, S., Chughtai, A. A., Fisman, D., Kunasekaran, M., Kvalsvig, A., Lupton, D., & Oliver, M. (2024). Masks and respirators for prevention of respiratory infections: a state of the science review. *Clinical microbiology reviews*, *37*(2), e00124-00123.

Hajmohammadi, M., Saki Malehi, A., & Maraghi, E. (2023). Effectiveness of Using Face Masks and Personal Protective Equipment to Reducing the Spread of COVID-19: A Systematic Review and Meta-Analysis of Case-Control Studies. *Adv Biomed Res*, *12*, 36. <https://doi.org/10.4103/abr.abr_337_21>

Iannone, P., Castellini, G., Coclite, D., Napoletano, A., Fauci, A. J., Iacorossi, L., D'Angelo, D., Renzi, C., La Torre, G., Mastroianni, C. M., & Gianola, S. (2020). The need of health policy perspective to protect Healthcare Workers during COVID-19 pandemic. A GRADE rapid review on the N95 respirators effectiveness. *PloS one*, *15*(6), e0234025. <https://doi.org/10.1371/journal.pone.0234025>

Jefferson, T., Dooley, L., Ferroni, E., Al-Ansary, L. A., van Driel, M. L., Bawazeer, G. A., Jones, M. A., Hoffmann, T. C., Clark, J., Beller, E. M., Glasziou, P. P., & Conly, J. M. (2023). Physical interventions to interrupt or reduce the spread of respiratory viruses. *Cochrane Database Syst Rev*, *1*(1), Cd006207. <https://doi.org/10.1002/14651858.CD006207.pub6>

Jefferson, T., Foxlee, R., Del Mar, C., Dooley, L., Ferroni, E., Hewak, B., Prabhala, A., Nair, S., & Rivetti, A. (2008). Physical interventions to interrupt or reduce the spread of respiratory viruses: systematic review. *BMJ*, *336*(7635), 77-80.

Jefferson, T., Jones, M., Al Ansari, L. A., Bawazeer, G., Beller, E., Clark, J., Conly, J., Del Mar, C., Dooley, E., & Ferroni, E. (2020). Physical interventions to interrupt or reduce the spread of respiratory viruses. Part 1-Face masks, eye protection and person distancing: systematic review and meta-analysis. *Cochrane Database of Systematic Reviews*, *11*.

Juneau, C. E., Pueyo, T., Bell, M., Gee, G., Collazzo, P., & Potvin, L. (2022). Lessons from past pandemics: a systematic review of evidence-based, cost-effective interventions to suppress COVID-19. *Syst Rev*, *11*(1), 90. <https://doi.org/10.1186/s13643-022-01958-9>

Kim, M. S., Seong, D., Li, H., Chung, S. K., Park, Y., Lee, M., Lee, S. W., Yon, D. K., Kim, J. H., Lee, K. H., Solmi, M., Dragioti, E., Koyanagi, A., Jacob, L., Kronbichler, A., Tizaoui, K., Cargnin, S., Terrazzino, S., Hong, S. H.,…Smith, L. (2022). Comparative effectiveness of N95, surgical or medical, and non-medical facemasks in protection against respiratory virus infection: A systematic review and network meta-analysis. *Rev Med Virol*, *32*(5), e2336. <https://doi.org/10.1002/rmv.2336>

Kunstler, B., Newton, S., Hill, H., Ferguson, J., Hore, P., Mitchell, B. G., Dempsey, K., Stewardson, A. J., Friedman, D., Cole, K., Sim, M. R., Ferguson, B., Burns, P., King, N., McGloughlin, S., Dicks, M., McCarthy, S., Tam, B., Hazelton, B.,…Turner, T. (2022). P2/N95 respirators & surgical masks to prevent SARS-CoV-2 infection: Effectiveness & adverse effects. *Infect Dis Health*, *27*(2), 81-95. <https://doi.org/10.1016/j.idh.2022.01.001>

Li, H., Yuan, K., Sun, Y. K., Zheng, Y. B., Xu, Y. Y., Su, S. Z., Zhang, Y. X., Zhong, Y., Wang, Y. J., Tian, S. S., Gong, Y. M., Fan, T. T., Lin, X., Gobat, N., Wong, S. Y. S., Chan, E. Y. Y., Yan, W., Sun, S. W., Ran, M. S.,…Lu, L. (2022). Efficacy and practice of facemask use in general population: a systematic review and meta-analysis. *Transl Psychiatry*, *12*(1), 49. <https://doi.org/10.1038/s41398-022-01814-3>

Li, J., Qiu, Y., Zhang, Y., Gong, X., He, Y., Yue, P., Zheng, X., Liu, L., Liao, H., Zhou, K., Hua, Y., & Li, Y. (2021). Protective efficient comparisons among all kinds of respirators and masks for health-care workers against respiratory viruses: A PRISMA-compliant network meta-analysis. *Medicine (Baltimore)*, *100*(34), e27026. <https://doi.org/10.1097/md.0000000000027026>

Li, Y., Liang, M., Gao, L., Ayaz Ahmed, M., Uy, J. P., Cheng, C., Zhou, Q., & Sun, C. (2021). Face masks to prevent transmission of COVID-19: A systematic review and meta-analysis. *Am J Infect Control*, *49*(7), 900-906. <https://doi.org/10.1016/j.ajic.2020.12.007>

Li, Y., Wei, Z., Zhang, J., Li, R., Li, H., Cao, L., Hou, L., Zhang, W., Chen, N., & Guo, K. (2021). Wearing masks to reduce the spread of respiratory viruses: a systematic evidence mapping. *Annals of Translational Medicine*, *9*(9), 811.

Liang, M., Gao, L., Cheng, C., Zhou, Q., Uy, J. P., Heiner, K., & Sun, C. (2020). Efficacy of face mask in preventing respiratory virus transmission: A systematic review and meta-analysis. *Travel Med Infect Dis*, *36*, 101751. <https://doi.org/10.1016/j.tmaid.2020.101751>

Long, Y., Hu, T., Liu, L., Chen, R., Guo, Q., Yang, L., Cheng, Y., Huang, J., & Du, L. (2020). Effectiveness of N95 respirators versus surgical masks against influenza: A systematic review and meta‐analysis. *Journal of Evidence‐Based Medicine*.

Lu, Y., Okpani, A. I., McLeod, C. B., Grant, J. M., & Yassi, A. (2023). Masking strategy to protect healthcare workers from COVID-19: An umbrella meta-analysis. *Infect Dis Health*. <https://doi.org/10.1016/j.idh.2023.01.004>

MacIntyre, C. R., & Chughtai, A. A. (2020). A rapid systematic review of the efficacy of face masks and respirators against coronaviruses and other respiratory transmissible viruses for the community, healthcare workers and sick patients. *Int J Nurs Stud*, *108*, 103629. <https://doi.org/10.1016/j.ijnurstu.2020.103629>

Mendez-Brito, A., El Bcheraoui, C., & Pozo-Martin, F. (2021). Systematic review of empirical studies comparing the effectiveness of non-pharmaceutical interventions against COVID-19. *J Infect*, *83*(3), 281-293. <https://doi.org/10.1016/j.jinf.2021.06.018>

Nanda, A., Hung, I., Kwong, A., Man, V. C., Roy, P., Davies, L., & Douek, M. (2021). Efficacy of surgical masks or cloth masks in the prevention of viral transmission: Systematic review, meta-analysis, and proposal for future trial. *J Evid Based Med*, *14*(2), 97-111. <https://doi.org/10.1111/jebm.12424>

Offeddu, V., Yung, C. F., Low, M. S. F., & Tam, C. C. (2017). Effectiveness of masks and respirators against respiratory infections in healthcare workers: a systematic review and meta-analysis. *Clinical Infectious Diseases*, *65*(11), 1934-1942.

Ollila, H., Partinen, M., Koskela, J., Borghi, J., Savolainen, R., Rotkirch, A., & Laine, L. T. (2022). Face masks to prevent transmission of respiratory infections: Systematic review and meta-analysis of randomized controlled trials on face mask use. *PloS one*, *17*(12), e0271517.

Peccin, M. S., Duarte, M. L., Imoto, A. M., Taminato, M., Saconato, H., Puga, M. E., Franco, E. S. B., Camargo, E. B., Gottems, L. B. D., & Atallah Á, N. (2022). Indications for accurate and appropriate use of personal protective equipment for healthcare professionals. A systematic review. *Sao Paulo Med J*, *140*(1), 56-70. <https://doi.org/10.1590/1516-3180.2021.0128.R1.18052021>

Perski, O., Szinay, D., Corker, E., Shahab, L., West, R., & Michie, S. (2022). Interventions to increase personal protective behaviours to limit the spread of respiratory viruses: A rapid evidence review and meta‐analysis. *British Journal of Health Psychology*, *27*(1), 215-264.

Peters, J. A., & Farhadloo, M. (2023). The Effects of Non-Pharmaceutical Interventions on COVID-19 Cases, Hospitalizations, and Mortality: A Systematic Literature Review and Meta-Analysis. *AJPM Focus*, 100125. <https://doi.org/10.1016/j.focus.2023.100125>

Ramaraj, P., Super, J., Doyle, R., Aylwin, C., & Hettiaratchy, S. (2020). Triaging of respiratory protective equipment on the assumed risk of SARS-CoV-2 aerosol exposure in patient-facing healthcare workers delivering secondary care: a rapid review. *BMJ Open*, *10*(10), e040321. <https://doi.org/10.1136/bmjopen-2020-040321>

Rohde, D., Ahern, S., Clyne, B., Comber, L., Spillane, S., Walsh, K., Carty, P., Drummond, L., Boland, T., & Smith, S. (2020). Effectiveness of face masks worn in community settings at reducing the transmission of SARS-CoV-2: a rapid review [version 1; peer review: 1 approved with reservations]. *HRB Open Research*, *3*, 76 <https://doi.org/https://doi.org/10.12688/hrbopenres.13161.1>

Samaranayake, L. P., Fakhruddin, K. S., Ngo, H. C., Chang, J. W. W., & Panduwawala, C. (2020). The effectiveness and efficacy of respiratory protective equipment (RPE) in dentistry and other health care settings: a systematic review. *Acta Odontologica Scandinavica*, *78*(8), 626-639.

Sami, H., Firoze, S., Khan, P. A., Fatima, N., & Khan, H. M. (2023). Face masks for respiratory viral illness prevention in healthcare settings: a concise systemic review and meta-analysis. *Iran J Microbiol*, *15*(2), 181-188. <https://doi.org/10.18502/ijm.v15i2.12466>

Schoberer, D., Osmancevic, S., Reiter, L., Thonhofer, N., & Hoedl, M. (2022). Rapid review and meta-analysis of the effectiveness of personal protective equipment for healthcare workers during the COVID-19 pandemic. *Public Health Pract (Oxf)*, *4*, 100280. <https://doi.org/10.1016/j.puhip.2022.100280>

SeyedAlinaghi, S., Karimi, A., Afsahi, A. M., Mirzapour, P., Varshochi, S., Mojdeganlou, H., Mojdeganlou, P., Razi, A., Alilou, S., Dashti, M., Ghasemzadeh, A., Saeidi, S., Mehraeen, E., & Dadras, O. (2023). The Effectiveness of Face Masks in Preventing COVID-19 Transmission: A Systematic Review. *Infect Disord Drug Targets*. <https://doi.org/10.2174/1871526523666230601090905>

Sharma, S. K., Mishra, M., & Mudgal, S. K. (2020). Efficacy of cloth face mask in prevention of novel coronavirus infection transmission: A systematic review and meta-analysis. *J Educ Health Promot*, *9*, 192. <https://doi.org/10.4103/jehp.jehp_533_20>

Smith, J., MacDougall, C. C., Johnstone, J., Copes, R. A., Schwartz, B., & Garber, G. E. (2016). Effectiveness of N95 respirators versus surgical masks in protecting health care workers from acute respiratory infection: a systematic review and meta-analysis. *Cmaj*, *188*(8), 567-574.

Smith, S. M., Sonego, S., Wallen, G. R., Waterer, G., Cheng, A. C., & Thompson, P. (2015). Use of non‐pharmaceutical interventions to reduce the transmission of influenza in adults: A systematic review. *Respirology*, *20*(6), 896-903.

Soleman, S. R., Lyu, Z., Okada, T., Sassa, M. H., Fujii, Y., Mahmoud, M. A., Ebner, D. K., & Harada, K. H. (2023). Efficacy of personal protective equipment to prevent environmental infection of COVID-19 among healthcare workers: a systematic review. *Environmental Health and Preventive Medicine*, *28*, 1-1.

Tabatabaeizadeh, S. A. (2021). Airborne transmission of COVID-19 and the role of face mask to prevent it: a systematic review and meta-analysis. *Eur J Med Res*, *26*(1), 1. <https://doi.org/10.1186/s40001-020-00475-6>

Talic, S., Shah, S., Wild, H., Gasevic, D., Maharaj, A., Ademi, Z., Li, X., Xu, W., Mesa-Eguiagaray, I., & Rostron, J. (2021). Effectiveness of public health measures in reducing the incidence of covid-19, SARS-CoV-2 transmission, and covid-19 mortality: systematic review and meta-analysis. *BMJ*, *375*.

Tran, T., Mostafa, E. M., Tawfik, G. M., Soliman, M., Mahabir, S., Mahabir, R., Dong, V., Ravikulan, R., Alhijazeen, S., & Farrag, D. A. (2021). Efficacy of face masks against respiratory infectious diseases: a systematic review and network analysis of randomized-controlled trials. *Journal of breath research*, *15*(4), 047102.

Wang, M. X., Gwee, S. X. W., Chua, P. E. Y., & Pang, J. (2020). Effectiveness of Surgical Face Masks in Reducing Acute Respiratory Infections in Non-Healthcare Settings: A Systematic Review and Meta-Analysis. *Front Med (Lausanne)*, *7*, 564280. <https://doi.org/10.3389/fmed.2020.564280>

Xiao, J., Shiu, E. Y. C., Gao, H., Wong, J. Y., Fong, M. W., Ryu, S., & Cowling, B. J. (2020). Nonpharmaceutical Measures for Pandemic Influenza in Nonhealthcare Settings-Personal Protective and Environmental Measures. *Emerg Infect Dis*, *26*(5), 967-975. <https://doi.org/10.3201/eid2605.190994>

Yin, X., Wang, X., Xu, S., & He, C. (2021). Comparative efficacy of respiratory personal protective equipment against viral respiratory infectious diseases in healthcare workers: a network meta-analysis. *Public Health*, *190*, 82-88.
