# Supplementary material for: Synthesis challenges in complex evidence: A critical analysis of systematic reviews of face mask efficacy
Source: Res Synth Methods. 2026 Feb 6;17(4):714–33. doi: 10.1017/rsm.2026.10072 (PMC13311340; doi:10.1017/rsm.2026.10072)
Supplement: Greenhalgh et al. supplementary material 2 — Greenhalgh et al. supplementary material [file S1759287926100726sup002.docx]

‘Tracker’ observational studies covered in systematic reviews

The five primary studies selected as tracker studies for the comparative analysis of these reviews consisted of:

- **Doung-Ngern 2020**:^1^ a community-based matched case-control study from Thailand during the COVID-19 pandemic, based on the contacts of people who tested positive (medical and non-medical masks);
- **Loeb 2004**:^2^ a hospital-based retrospective cohort study from Canada during the SARS outbreak, looking at nurses exposed to SARS patients (medical masks and N95s);
- **Seto 2003**:^3^ a hospital-based unmatched case-control study from Hong Kong during the SARS outbreak, looking at healthcare workers exposed to SARS patients (medical masks and N95s);
- **Wang Y 2020**:^4^ a community-based outbreak analysis (retrospective cohort design) from China during the COVID-19 pandemic looking at secondary infections in households (mask type not specified);
- **Zhang 2013**:^5^ an unmatched case-control study of a plane flight between USA, Hong Kong and China looking at influenza transmission from a single index case (mask type not specified)

# Summary of how these studies featured in meta-analyses

| TABLE S1: HOW THE TRACKER STUDIES WERE INCLUDED IN META-ANALYSES | | | | | |
| --- | --- | --- | --- | --- | --- |
| Meta-analysis  by | Tracker primary study | | | | |
|  | Doung-Ngern 2020^1^ (COVID-19, community, case-control) | Loeb 2004^2^ (SARS, healthcare, retrospective cohort) | Seto 2003^3^ (SARS, healthcare, case-control) | Wang Y 2020^4^ (COVID-19, community, retrospective cohort) | Zhang 2013^5^ (influenza, in-flight transmission, case-control) |
| Talic 2021^6^ (public health measures for COVID-19, hence community settings only) | One of 6 studies (including RCT & observational), all in COVID-19, produced RR 0.47 (0.29-0.75) | Not included (out of scope, not COVID-19, not community) | Not included (out of scope, not COVID-19, not community) | One of 6 studies (including RCT & observational), all in COVID-19, produced RR 0.47 (0.29-0.75) | Not included (out of scope, not COVID-19) |
| Chen 2022^7^ (masks against respiratory viral infections, healthcare and community) | One of 18 case-control studies of viral respiratory infections, produced RR 0.36 (0.26-0.28) | One of 7 cohort studies of viral respiratory infections, produced RR 0.31 (0.22-0.44) | One of 18 case-control studies of viral respiratory infections, produced RR 0.36 (0.26-0.28) | One of 7 cohort studies of viral respiratory infections, produced RR 0.31 (0.22-0.44) | One of 18 case-control studies of viral respiratory infections, produced RR 0.36 (0.26-0.28) |
| Kim 2022^8^ (comparative effectiveness of N95, medical and non‐medical masks against respiratory virus infection in healthcare and community) | Included in network meta-analysis, one of 8 studies of any mask in SARS, MERS or COVID-19, OR 0.41 (0.31-0.78), plus (separately) in sub-analyses of mask types in COVID-19 (medical mask, 6 studies and non-medical mask, 2 studies) | Included in network meta-analysis, one of 8 studies of any mask in SARS, MERS or COVID-19, OR 0.41 (0.31-0.78), plus (separately) in sub-analyses of mask types in SARS (N95, 4 studies and medical mask, 3 studies) | Included in network meta-analysis, one of 8 studies of any mask in SARS, MERS or COVID-19, OR 0.41 (0.31-0.78), plus (separately) in sub-analyses of mask types in SARS (N95, 4 studies and medical mask, 3 studies) | Not included (study was not identified by reviewers) | Not included (study was not identified by reviewers) |
| Hajmohammadi 2023^9^ (masks and other interventions to reduce spread of COVID-19, healthcare and community) | Included in two separate meta-analyses: one of 6 Asian studies (all designs), produced OR 0.45 (0.27-0.78), plus one of 6 studies on non-HCWs (all designs), produced OR 0.58 (0.31-1.06) | Not included (out of scope, not COVID-19) | Not included (out of scope, not COVID-19) | Not included (study was not identified by reviewers) | Not included (out of scope, not COVID-19) |
| Floriano 2024^10^ (masking to prevent COVID-19 infection in the community, observational studies only) | Included as one of 6 case-control studies of medical masks in COVID-19, producing OR 0.51 (0.37-0.70) | Included as one of 2 cohort studies of medical masks in COVID-19, producing a risk difference of  -0.21 (-0.24 to -0.07) | Not included (out of scope, not COVID-19) | Not included (study was not identified by reviewers) | Not included (out of scope, not COVID-19) |
| Tabatabaeizadeh 2021^11^ (masks in preventing airborne transmission of SARS-CoV-2) | Included as one of 4 studies of masks in COVID-19 (all designs), producing OR 0.12 (0.06-0.27) | Not included (study was not identified by reviewer) | Not included (out of scope, not COVID-19) | Not included (study was not identified by reviewer, perhaps not yet published) | Not included (out of scope, not COVID-19) |
| Wang M 2020^12^ (surgical masks for prevention of respiratory infections in non-healthcare settings) | Not included (study was not identified by reviewers) | Not included (out of scope, healthcare setting) | Not included (out of scope, healthcare setting) | Not included (study was not identified by reviewers) | Included as the sole study in a meta-analysis of in-flight transmission of acute respiratory infections, OR 0.06 (0 – 1.11) |
| Li Y 2021^13^ (masks against COVID-19 infection, healthcare and community settings) | Included in two separate meta-analyses of masks in COVID-19: one of 6 observational studies, producing OR 0.38 (0.21-0.69), and the sole study in a sub-analysis of non-healthcare settings, oddly using unadjusted rather than adjusted result, producing OR 0.72 (0.46-1.12) | Not included (out of scope, not COVID-19) | Not included (out of scope, not COVID-19) | Included in two separate meta-analyses of masks in COVID-19: one of 6 observational studies, producing OR 0.38 (0.21-0.69), and one of 5 studies in a sub-analysis of healthcare settings, producing OR 0.29 (0.18-0.44) | Not included (out of scope, not COVID-19) |
| Chu 2020^14^ (physical interventions against respiratory coronaviruses (SARS, MERS, SARS-CoV-2). HCW and community) | Not included (study was not identified by reviewers) | Included as one of 26 observational studies (all kinds) in meta-analysis of masks in SARS, MERS and COVID-19 in healthcare workers, producing OR 0.30 (0.22-0.41), plus in separate analysis, one of 17 observational studies (all types) of masks in control of SARS, producing OR 0.35 (0.23-0.52). Also included in various supplementary analyses. | Included as one of 26 observational studies (all kinds) in meta-analysis of masks in SARS, MERS and COVID-19 in healthcare workers, producing OR 0.3 (0.22-0.41), plus as one of 4 observational studies of N95s vs no mask, OR 0.04 (0.004-0.030). Also included in various supplementary analyses. | Not included (study was not identified by reviewer, perhaps not yet published) | Not included (study was not identified by reviewers) |
| Li J 2021^15^ (respirators and masks against respiratory infection in healthcare workers, network meta-analysis) | Not included (out of scope, not healthcare) | Included in network meta-analysis for N95 vs medical masks (number of studies not stated), producing OR 0.43 (0.02-0.94) | Not included (study was not identified by reviewers) | Not included (out of scope, not healthcare) | Not included (out of scope, not healthcare) |
| Liang 2020^16^ (respirators versus masks, any infection, healthcare and community) | Not included (study was not identified by reviewers) | Included as one of 21 studies (RCT plus observational) in meta-analysis of masks in control of viral respiratory infections, producing OR 0.35 (0.24-0.51) | Not included (study was not identified by reviewers) | Not included (study was not identified by reviewers) | Included as one of 21 studies (RCT plus observational) in meta-analysis of masks in control of viral respiratory infections, producing OR 0.35 (0.24-0.51) |
| Smith 2016^17^ (respirators v masks for prevention of infections in healthcare workers) | Not included (out of scope – not N95 vs masks) | Included as the sole study in meta-analysis of cohort studies of N95s v medical masks, producing OR 0.43 (0.03–6.41) | Included as one of two studies in meta-analysis of case-control studies of N95s vs medical masks, producing OR 0.91 (0.25–3.36) | Not included (out of scope – not N95 vs masks) | Not included (out of scope – not N95 vs masks) |
| Jefferson 2008^18^ (physical interventions to reduce spread of respiratory infections) | Not yet published when review done | Not included (study was not identified by reviewers) | Included as 1 of 6 observational studies in meta-analysis of masks vs control (both healthcare and community), producing OR 0.32 (0.25 to 0.40) | Not yet published when review done | Not yet published when review done |
| Offeddu 2017^19^ (masks and respirators for healthcare worker protection against respiratory infection) | Not yet published when review done and out of scope anyway | Included as 1 of 16 observational studies (healthcare settings only) of any mask vs no mask in prevention of respiratory infections, producing OR 0.22 (0.12-0.40), plus as 1 of 5 observational studies in analysis of medical mask v no mask (OR 0.13 (0.03-0.62)), 5 studies of N95 v no mask (OR 0.12 (0.06-0.26)), and 2 studies of N95 v medical masks (OR 0.86 (0.22-3.33)) | Included as 1 of 16 observational studies (healthcare settings only) of any mask vs no mask in prevention of respiratory infections, producing OR 0.22 (0.12-0.40), plus as 1 of 5 observational studies in analysis of medical mask v no mask (OR 0.13 (0.03-0.62)), and 5 studies of N95 v no mask (OR 0.12 (0.06-0.26)). | Not yet published when review done | Out of scope (not healthcare) |
| Sami 2023^20^ (masks for viral illness prevention in healthcare settings) | Out of scope (not healthcare) | Not included (study was not identified by reviewers) | Included as 1 of 6 observational studies in meta-analysis of masks in prevention of respiratory viral infections in healthcare workers, producing OR 0.11 (0.04-0.33) | Out of scope (not healthcare) | Out of scope (not healthcare) |
| Coclite 2021^21^ (effectiveness of face masks in the community to prevent “COVID-19 or similar pandemic”) | Not included (study was not identified by reviewers) | Out of scope (healthcare setting) | Out of scope (healthcare setting) | Not included (study was not identified by reviewers) | Included as 1 of 4 studies in a meta-analysis of community-based case-control studies of masks in prevention of viral respiratory infections, producing OR 0.59 (0.34-1.03). The other studies included a pilgrim (mass gathering) study in which people shared tents |

# Doung-Ngern 2020^1^

ROB assessed in 11 systematic reviews.^6-11,13,22-25^ This example is described and discussed in the main paper; tables are reproduced here for completeness.

| TABLE S2: SCORES AWARDED BY SYSTEMATIC REVIEWS TO THE  DOUNG-NGERN STUDY USING THE NEWCASTLE-OTTAWA SCALE (NOS) | | | | |
| --- | --- | --- | --- | --- |
|  | Chen^7^ | Hajmohammedi^9^ | Li Y^13^ | Tabatabaeizadeh^11^ |
| SELECTION |  |  |  |  |
| Adequacy of case definition | * | Not stated | * | Not stated |
| Representativeness of cases | * | Not stated | * | Not stated |
| Selection of controls | * | Not stated | * | Not stated |
| Definition of controls | * | Not stated | * | Not stated |
| COMPARABILITY |  |  |  |  |
| Key confounders controlled for? | ** | Not stated | ** | Not stated |
| EXPOSURE |  |  |  |  |
| How exposure was ascertained | * | Not stated | * | Not stated |
| Same for cases and controls? | * | Not stated | * | Not stated |
| Losses to follow-up similar in both groups? | * | Not stated | * | Not stated |
| TOTAL SCORE | 9/9 | 8/9 | 9/9 | Not stated |
| Narrative | None | None | None | None. |
| Notes |  | Only total score given, no breakdown by domain. Emailed author 22.9.25 |  | Authors claim they used NOS but no results given. Emailed author 22.9.25 |

| TABLE S3: SCORES AWARDED BY A SYSTEMATIC REVIEW TO THE  DOUNG-NGERN STUDY USING JOANNA BRIGGS INSTITUTE CHECKLIST^26^ | | | |
| --- | --- | --- | --- |
|  | Crespo^24^ | Rohde^25^ |  |
| 1. Were the groups comparable other than the presence of disease in cases or the absence of disease in controls? | Low (i.e. low  risk of bias, answer to question is yes) | Yes |  |
| 1. Were cases and controls matched appropriately? | Low (i.e. yes) | No |  |
| 1. Were the same criteria used for identification of cases and controls? | Low (i.e. yes) | Yes |  |
| 1. Was exposure measured in a standard, valid and reliable way? | Low (i.e. yes) | No |  |
| 1. Was exposure measured in the same way for cases and controls? | Low (i.e. yes) | Yes |  |
| 1. Were confounding factors identified? | Low (i.e. yes) | Unclear |  |
| 1. Were strategies to deal with confounding factors stated? | Low (i.e. yes) | Unclear |  |
| 1. Were outcomes assessed in a standard, valid and reliable way for cases and controls? | Low (i.e. yes) | Unclear |  |
| 1. Was the exposure period of interest long enough to be meaningful? | Low (i.e. yes) | Yes |  |
| 1. Was appropriate statistical analysis used? | Low (i.e. yes) | Unclear |  |
| OVERALL ASSESSMENT | Low risk of bias | Not stated |  |
| Narrative | None | “Outcomes based on self-report, not all controls were PCR tested (some may have been asymptomatic)” |  |
| Notes |  | Abstract states “all studies were at high risk of bias” but no further information is given in text; raw scores obtained from the authors |  |

| TABLE S4: SCORES AWARDED BY A SYSTEMATIC REVIEW TO THE DOUNG-NGERN STUDY USING US PREVENTIVE SERVICES TASKFORCE CHECKLIST^27^ | |
| --- | --- |
|  | Chou^23^ |
| 1. Did the study attempt to enrol all (or a random sample of) patients meeting inclusion criteria (inception cohort)? | Yes |
| 1. Did the study use accurate methods for ascertaining exposures and potential confounders? | No (likely recall bias) |
| 1. Were outcome assessors and/or data analysts blinded to exposure being studied? | Unclear |
| 1. Did the article report attrition or missing data? | No |
| 1. Is there high attrition or missing data? | Yes |
| 1. Were outcomes pre-specified and defined, and ascertained using accurate methods? | Yes |
| 1. Controlled for confounders?* | Yes |
| OVERALL ASSESSMENT | POOR |
| Narrative | Just the annotations above |

*To be graded “yes” studies had to control at a minimum for exposures and behaviours (e.g., other infection control measures).

| TABLE S5: SCORES AWARDED BY SYSTEMATIC REVIEWS TO THE  DOUNG-NGERN STUDY USING ROBINS-I | | | |
| --- | --- | --- | --- |
|  | Floriano^10^ | Kim^8^ | Talic^6^ |
| 1. Confounding | low | low | low |
| 1. Selection of participants | moderate | low | serious |
| 1. Classification of interventions | low | low | moderate |
| 1. Deviations from intended interventions | moderate | moderate | serious |
| 1. Missing data | serious | low | low |
| 1. Measurement of outcomes | low | low | low |
| 1. Selection of reported results | low | low | low |
| OVERALL ASSESSMENT | serious | moderate | serious |
| Narrative | None | See below | See below |
| Notes | No supplementary materials | No overall score awarded but tool rules say lowest-scoring domain determines score |  |

| TABLE S5a:  Full comment from Kim et al on Doung-Ngern (from supplementary file page 52-53) | | |
| --- | --- | --- |
| Bias | Author’s judgment | Support for judgment |
| **Pre-intervention domains** | | |
| Bias due to confounding  (Confounding) | Low risk | Case control study. Multivariable logistic regression models used to estimate associations between diagnosis of COVID-19 and covariates. Multivariate analysis that adjusted for major variables. |
| Bias in selection of participants into the study  (Selection bias) | Low risk | 211 cases and 839 non-matched controls using all contact tracing records of Thailand’s national Surveillance and Rapid Response Team. All patients in the enrolment period were screened for eligibility. |
| **At-intervention domain** | | |
| Bias in classification of interventions  (Information bias) | Low risk | Patients were categorized as case group and control group. Cases were asymptomatic contacts of COVID-19 patients diagnosed with COVID-19 in a specified duration of time. Controls were asymptomatic contacts who were not diagnosed with COVID-19. |
| **Post-intervention domains** | | |
| Bias due to deviations from intended interventions  (Confounding) | Moderate risk | This study did not evaluate the probability of contact with other infected people in the community setting. Cases with mild/no symptoms could have been missed. Also, impossible to identify every potential contact with and some may have had contact with more than one COVID-19 patient. |
| Bias due to missing data  (Selection bias) | Low risk | Missing values for mark wearing not included in analyses. For other variables, missing values were random and were imputed by chained equations. |
| Bias in measurement of the outcome  (Information bias) | Low risk | Assessment of outcome may be affected because findings subject to memory bias, observer bias and information bias. To reduce potential bias, structured interviews used where each participant was asked the same set of defined questions. |
| Bias in selection of the reported result  (Reporting bias) | Low risk | A STROBE statement checklist provided that give evidence of transparent reporting. |

Talic et al comment on Doung-Ngern (supplementary material page 54): “Findings based on contacts of cases, not generalizable to all settings. Studies do not evaluate the probability of contact with a different case from the index case. Only 90% of controls were tested so some of them might have been cases. Not all contacts were identified.”

# Loeb 2004^2^

Loeb 2004 was ROB assessed by 7 systematic reviews (6 NOS^7,14-17,19^ and one ROBINS-I^8^):

| TABLE S6: SCORES AWARDED BY SYSTEMATIC REVIEWS TO THE  LOEB 2004 STUDY USING THE NEWCASTLE-OTTAWA SCALE (NOS) | | | | | | |
| --- | --- | --- | --- | --- | --- | --- |
|  | Chen^7^ | Chu^14^ | Li J^15^ | Liang^16^ | Offeddu^19^ | Smith 2016^17^ |
| SELECTION |  |  |  |  |  |  |
| Adequacy of case definition | Total 5 stars given (the award above the maximum is not explained) | Total 2 stars given (sub-domains not stated) | * | Total 3 stars given (sub-domains not stated) | * | 0 |
| Representativeness of cases |  |  | * |  | * | * |
| Selection of controls |  |  | * |  | * | * |
| Definition of controls |  |  | 0 |  | * | * |
| COMPARABILITY |  |  |  |  |  |  |
| Key confounders controlled for? | ** | 0 | ** | ** | 0 | 0 |
| EXPOSURE |  |  |  |  |  |  |
| How exposure was ascertained | 0 | 0 | 0 | Total 2 stars given (sub-domains not stated) | * | * |
| Same for cases and controls? | 0 | 0 | * |  | * | * |
| Losses to follow-up similar in both groups? | 0 | 0 | * |  | * | * |
| TOTAL SCORE | 7/9 | 2/9 | 7/9 | 7/9 | 7/9 | 6/9 |
| Narrative | None | None | None | None | “Potential recall bias, although minimized by corroboration with medical records; small sample size.” | None |

| TABLE S7: SCORES AWARDED BY SYSTEMATIC REVIEWS TO THE  LOEB 2004 STUDY USING ROBINS-I | | | |
| --- | --- | --- | --- |
|  | Kim^8^ | Notes from Kim et al in “support for judgement” column | |
| 1. Confounding | serious | Retrospective cohort study. Baseline characteristics were generally comparable except age. Also, potential for unknown confounders remains. | |
| 1. Selection of participants | low | Forty-three nurses worked at least one shift in a critical care unit where there was a patient with SARS were enrolled. Start of intervention and follow-up coincide. | |
| 1. Classification of interventions | low | Intervention definition was clear and based solely on information collected at the time of intervention. | |
| 1. Deviations from intended interventions | low | Deviations are unlikely, and deviations that do occur are likely to reflect usual clinical practice. | |
| 1. Missing data | low | Data were complete | |
| 1. Measurement of outcomes | moderate | They minimized bias using medical records. However, Certain outcome measures (e.g. personal protective equipment) may be influenced by knowledge of intervention received by patients. | |
| 1. Selection of reported results | “no information” | Unclear if authors selected this outcome measure a priori | |
| OVERALL ASSESSMENT | Not stated but serious risk of bias implied from lowest-scoring domain |  |  |

# Seto 2003^3^

Seto 2003 is assessed for ROB in 8 reviews^7,8,14,15,17-20^ (7 NOS and one ROBINS-I).

| TABLE S8: SCORES AWARDED BY SYSTEMATIC REVIEWS TO THE  SETO STUDY USING THE NEWCASTLE-OTTAWA SCALE (NOS) | | | | | | | |
| --- | --- | --- | --- | --- | --- | --- | --- |
|  | Chen^7^ | Chu^14^ | Jefferson 2008^18^ | Li J^15^ | Offeddu^19^ | Sami^20^ | Smith 2016^17^ |
| SELECTION |  |  |  |  |  |  |  |
| Adequacy of case definition | Total 2 stars given (sub-domains not stated) | * | Not stated | * | * | * | 0 |
| Representativeness of cases |  | * | Not stated | * | * | * | 0 |
| Selection of controls |  | * | Not stated | * | * | * | * |
| Definition of controls |  | * | Not stated | 0 | * | * | * |
| COMPARABILITY |  |  |  |  |  |  |  |
| Key confounders controlled for? | * | ** | Not stated | ** | ** | ** | 0 |
| EXPOSURE |  |  |  |  |  |  |  |
| How exposure was ascertained | Total 1 star given (sub-domain not stated) | Total 2 stars given (sub-domains not stated) | Not stated | 0 | Total 2 stars given (sub-domains not stated) | Total 2 stars given (sub-domains not stated) | 0 |
| Same for cases and controls? |  |  | Not stated | * |  |  | * |
| Losses to follow-up similar in both groups? |  |  | Not stated | * |  |  | 0 |
| TOTAL SCORE | 4/9 | 8/9 | “Medium” | 7/9 | 8/9 | 8/9 | 3/9 |
| Narrative | None | None | “inconsistencies in text: lack of description of controls” | None | “Potential recall bias, although minimized by corroboration with medical records; small sample size.” |  |  |

| TABLE S9: SCORES AWARDED BY SYSTEMATIC REVIEWS TO THE  SETO STUDY USING ROBINS-I | | |
| --- | --- | --- |
|  | Kim^8^ | Notes from Kim et al in “support for judgement” column |
| 1. Confounding | critical | Case-control study; Baseline characteristics were not considered. |
| 1. Selection of participants | low | Infected hospital staff were those who acquired SARS 2–7 days after exposure, with no exposure to cases outside the hospital. They tested sera taken from index patients and infected hospital staff during the acute phase of the infection and during convalescence for antibodies to the corona-like virus 4 associated with SARS using an indirect immunoflourescence test. |
| 1. Classification of interventions | moderate | Interventions of interest are protective equipment during exposure to index patients, and were determined retrospectively. |
| 1. Deviations from intended interventions | low | Deviations from intended intervention likely reflect usual clinical practice. |
| 1. Missing data | moderate | 356 completed questionnaires were returned, covering 85% of the staff on roster. The analysis is unlikely to have removed the risk of bias arising from the missing data. |
| 1. Measurement of outcomes | moderate | Main outcome measure was infection with evidence of clinical manifestation and serology. Knowledge of intervention received may have minimally affected certain aspects of infection. |
| 1. Selection of reported results | “no information” | Unclear if authors selected the reported outcome measures a priori |
| OVERALL ASSESSMENT | Not stated but critical risk of bias implied from lowest-scoring domain |  |

# Wang Y^4^

ROB assessed in 5 systematic reviews,^6,7,10,25,28,29^ 2 with NOS, 3 with ROBINS-I and 1 with GRADE.

| TABLE S10: SCORES AWARDED BY SYSTEMATIC REVIEWS TO THE  WANG Y STUDY USING THE NEWCASTLE-OTTAWA SCALE (NOS) | | |
| --- | --- | --- |
|  | Chen^7^ | Rohde^25^ |
| SELECTION |  |  |
| Adequacy of case definition | No details given | No details given |
| Representativeness of cases |  |  |
| Selection of controls |  |  |
| Definition of controls |  |  |
| COMPARABILITY |  |  |
| Key confounders controlled for? | No details given | No details given |
| EXPOSURE |  |  |
| How exposure was ascertained | No details given | No details given |
| Same for cases and controls? |  |  |
| Losses to follow-up similar in both groups? |  |  |
| TOTAL SCORE | 5/9 | No score given |
| Narrative | None | Abstract states “all studies were at high risk of bias” |

| TABLE S11: SCORES AWARDED BY SYSTEMATIC REVIEWS TO THE  WANG Y STUDY USING ROBINS-I | | | |
| --- | --- | --- | --- |
|  | Floriano^10^ | Talic^6^ | Nanda^28^ |
| 1. Confounding | serious | low | serious |
| 1. Selection of participants | critical | serious | serious |
| 1. Classification of interventions | low | low | serious |
| 1. Deviations from intended interventions | low | critical | serious |
| 1. Missing data | low | critical | serious |
| 1. Measurement of outcomes | low | low | low |
| 1. Selection of reported results | low | moderate | serious |
| OVERALL ASSESSMENT | serious | moderate | serious |
| Narrative | None | None | None |

Chaabna et al claim to have used GRADE, but the actual application was as follows: “Randomized clinical trials were considered as high-level evidence (level 1), observational studies (such as cohort and case control studies) as low-level evidence (level 2), and any other evidence as very low evidence (level 3).” (page 199). As such, the Wang study was allocated a “low level evidence” rating.

# Zhang 2013^5^

ROB assessed by 7 systematic reviews: 6 using NOS^7,15-17,19,21^ and one using National Heart, Lung and Blood Institute quality assessment checklist.^12^

| TABLE S12: SCORES AWARDED BY SYSTEMATIC REVIEWS TO THE  ZHANG 2013 STUDY USING THE NEWCASTLE-OTTAWA SCALE (NOS) |
| --- |

|  | Chen^7^ | Coclite^21^ | Li J^15^ | Liang^16^ | Offeddu^19^ | Smith 2016^17^ |
| --- | --- | --- | --- | --- | --- | --- |
| SELECTION |  |  |  |  |  |  |
| Adequacy of case definition | * | * | 0 | * | * | 0 |
| Representativeness of cases | * | * | * | * | * | * |
| Selection of controls | * | * | * | * | * | * |
| Definition of controls | * | 0 | 0 | * | * | * |
| COMPARABILITY |  |  |  |  |  |  |
| Key confounders controlled for? | ** | * | ** | ** | ** | ** |
| EXPOSURE |  |  |  |  |  |  |
| How exposure was ascertained | Total 1 star given (sub-domain not stated) | * | 0 | Total 1 star given (sub-domain not stated) | Total 2 stars given (sub-domains not stated) | 0 |
| Same for cases and controls? |  | 0 | * |  |  | * |
| Losses to follow-up similar in both groups? |  | 0 | 0 |  |  | 0 |
| TOTAL SCORE | 7/9 | “fair” | 5/9 | 7/9 | 8/9 | 6/9 |
| Narrative | None | None | None | None | “Non-differential imperfect recall among cases or controls.  Controls might be more likely to have forgotten about high-risk behaviors than cases. Small sample size. Serological tests not performed on controls, may lead to under-estimated ORs. Matching procedure prevents analysis by type of occupational duties or type of ward. Factors outside hospital omitted.” | None |

| TABLE S12: SCORES AWARDED BY SYSTEMATIC REVIEW TO THE ZHANG 2013 STUDY USING NATIONAL HEART, LUNG AND BLOOD INSTITUTE CHECKLIST | |
| --- | --- |
|  | Wang 2020^12^ |
| 1. Selection | High (i.e. study is high quality in this domain) |
| 1. Misclassification | Low |
| 1. Detection | Low |
| 1. Confounding | High |
| 1. Other (inappropriate sample size, attrition) | Low |

1. Doung-Ngern P, Suphanchaimat R, Panjangampatthana A, et al. Case-control study of use of personal protective measures and risk for SARS-CoV 2 infection, Thailand. *Emerging infectious diseases*. 2020;26(11):2607.

2. Loeb M, McGeer A, Henry B, et al. SARS among critical care nurses, Toronto. *Emerg Infect Dis*. Feb 2004;10(2):251-5. doi:10.3201/eid1002.030838

3. Seto WH, Tsang D, Yung RW, et al. Effectiveness of precautions against droplets and contact in prevention of nosocomial transmission of severe acute respiratory syndrome (SARS). *Lancet*. May 3 2003;361(9368):1519-20. doi:10.1016/s0140-6736(03)13168-6

4. Wang Y, Tian H, Zhang L, et al. Reduction of secondary transmission of SARS-CoV-2 in households by face mask use, disinfection and social distancing: a cohort study in Beijing, China. *BMJ global health*. 2020;5(5):e002794.

5. Zhang L, Peng Z, Ou J, et al. Protection by face masks against influenza A(H1N1)pdm09 virus on trans-Pacific passenger aircraft, 2009. *Emerg Infect Dis*. 2013;19(9):1403-10. doi:10.3201/eid1909.121765

6. Talic S, Shah S, Wild H, et al. Effectiveness of public health measures in reducing the incidence of covid-19, SARS-CoV-2 transmission, and covid-19 mortality: systematic review and meta-analysis. *bmj*. 2021;375

7. Chen Y, Wang Y, Quan N, Yang J, Wu Y. Associations Between Wearing Masks and Respiratory Viral Infections: A Meta-Analysis and Systematic Review. *Front Public Health*. 2022;10:874693. doi:10.3389/fpubh.2022.874693

8. Kim MS, Seong D, Li H, et al. Comparative effectiveness of N95, surgical or medical, and non-medical facemasks in protection against respiratory virus infection: A systematic review and network meta-analysis. *Rev Med Virol*. Sep 2022;32(5):e2336. doi:10.1002/rmv.2336

9. Hajmohammadi M, Saki Malehi A, Maraghi E. Effectiveness of Using Face Masks and Personal Protective Equipment to Reducing the Spread of COVID-19: A Systematic Review and Meta-Analysis of Case-Control Studies. *Adv Biomed Res*. 2023;12:36. doi:10.4103/abr.abr_337_21

10. Floriano I, Silvinato A, Bacha HA, Barbosa AN, Tanni S, Bernardo WM. Effectiveness of wearing masks during the COVID-19 outbreak in cohort and case-control studies: a systematic review and meta-analysis. *Jornal Brasileiro de Pneumologia*. 2024;49:e20230003.

11. Tabatabaeizadeh SA. Airborne transmission of COVID-19 and the role of face mask to prevent it: a systematic review and meta-analysis. *Eur J Med Res*. Jan 2 2021;26(1):1. doi:10.1186/s40001-020-00475-6

12. Wang MX, Gwee SXW, Chua PEY, Pang J. Effectiveness of Surgical Face Masks in Reducing Acute Respiratory Infections in Non-Healthcare Settings: A Systematic Review and Meta-Analysis. *Front Med (Lausanne)*. 2020;7:564280. doi:10.3389/fmed.2020.564280

13. Li Y, Liang M, Gao L, et al. Face masks to prevent transmission of COVID-19: A systematic review and meta-analysis. *Am J Infect Control*. Jul 2021;49(7):900-906. doi:10.1016/j.ajic.2020.12.007

14. Chu DK, Akl EA, Duda S, et al. Physical distancing, face masks, and eye protection to prevent person-to-person transmission of SARS-CoV-2 and COVID-19: a systematic review and meta-analysis. *The Lancet*. 2020;395(10242):1973-1987. doi:10.1016/S0140-6736(20)31142-9

15. Li J, Qiu Y, Zhang Y, et al. Protective efficient comparisons among all kinds of respirators and masks for health-care workers against respiratory viruses: A PRISMA-compliant network meta-analysis. *Medicine (Baltimore)*. Aug 27 2021;100(34):e27026. doi:10.1097/md.0000000000027026

16. Liang M, Gao L, Cheng C, et al. Efficacy of face mask in preventing respiratory virus transmission: A systematic review and meta-analysis. *Travel Med Infect Dis*. Jul-Aug 2020;36:101751. doi:10.1016/j.tmaid.2020.101751

17. Smith J, MacDougall CC, Johnstone J, Copes RA, Schwartz B, Garber GE. Effectiveness of N95 respirators versus surgical masks in protecting health care workers from acute respiratory infection: a systematic review and meta-analysis. *Cmaj*. 2016;188(8):567-574.

18. Jefferson T, Foxlee R, Del Mar C, et al. Physical interventions to interrupt or reduce the spread of respiratory viruses: systematic review. *Bmj*. 2008;336(7635):77-80.

19. Offeddu V, Yung CF, Low MSF, Tam CC. Effectiveness of masks and respirators against respiratory infections in healthcare workers: a systematic review and meta-analysis. *Clinical Infectious Diseases*. 2017;65(11):1934-1942.

20. Sami H, Firoze S, Khan PA, Fatima N, Khan HM. Face masks for respiratory viral illness prevention in healthcare settings: a concise systemic review and meta-analysis. *Iran J Microbiol*. Apr 2023;15(2):181-188. doi:10.18502/ijm.v15i2.12466

21. Coclite D, Napoletano A, Gianola S, et al. Face mask use in the community for reducing the spread of COVID-19: a systematic review. *Frontiers in medicine*. 2021;7:594269.

22. Boulos L, Curran JA, Gallant A, et al. Effectiveness of face masks for reducing transmission of SARS-CoV-2: a rapid systematic review. *Philosophical Transactions of the Royal Society A*. 2023;381(2257):20230133.

23. Chou R, Dana T. Major Update: Masks for Prevention of SARS-CoV-2 in Health Care and Community Settings-Final Update of a Living, Rapid Review. *Ann Intern Med*. Jun 2023;176(6):827-835. doi:10.7326/m23-0570

24. Crespo N, Fornasier J, Dionicio P, Godino J, Ramers C, Elder J. Effectiveness of Face Masks in Preventing COVID-19 Transmission in Real-World Settings: A Systematic Literature Review. *Research Square*. 2023 2023;doi:10.21203/rs.3.rs-2534269/v1

25. Rohde D, Ahern S, Clyne B, et al. Effectiveness of face masks worn in community settings at reducing the transmission of SARS-CoV-2: a rapid review [version 1; peer review: 1 approved with reservations]. *HRB Open Research*. 2020;3:76 doi:<https://doi.org/10.12688/hrbopenres.13161.1>

26. Aromataris E, Stern C, Lockwood C, et al. JBI series paper 2: tailored evidence synthesis approaches are required to answer diverse questions: a pragmatic evidence synthesis toolkit from JBI. *Journal of clinical epidemiology*. 2022;150:196-202.

27. US Preventive Services Task Force. *Procedure Manual, Appendix VI. Criteria for Assessing Internal Validity of Individual Studies*. USPSTF. Accessed 24th September 2025 at <https://www.uspreventiveservicestaskforce.org/uspstf/about-uspstf/methods-and-processes/procedure-manual/procedure-manual-appendix-vi-criteria-assessing-internal-validity-individual-studies>; 2017.

28. Nanda A, Hung I, Kwong A, et al. Efficacy of surgical masks or cloth masks in the prevention of viral transmission: Systematic review, meta-analysis, and proposal for future trial. *J Evid Based Med*. May 2021;14(2):97-111. doi:10.1111/jebm.12424

29. Chaabna K, Doraiswamy S, Mamtani R, Cheema S. Facemask use in community settings to prevent respiratory infection transmission: A rapid review and meta-analysis. *International Journal of Infectious Diseases*. 2021;104:198-206.
